# Circ-SIRT1 inhibits cardiac hypertrophy via activating SIRT1 to promote autophagy

**DOI:** 10.1038/s41419-021-04059-y

**Published:** 2021-11-10

**Authors:** Weichen Wang, Longlong Wang, Mengyue Yang, Chunwei Wu, Rui Lan, Weiwei Wang, Yuze Li

**Affiliations:** Department of Cardiology, the First Hospital of China Medical University, 110001 Shenyang, Liaoning People’s Republic of China

**Keywords:** Biotechnology, Cell biology

## Abstract

Mounting studies have substantiated that abrogating autophagy contributes to cardiac hypertrophy (CH). Sirtuin 1 (SIRT1) has been reported to support autophagy and inhibit CH. However, the upstream regulation mechanism behind the regulation of SIRT1 level in CH remains unclear. Circular RNAs (circRNAs) are vital modulators in diverse human diseases including CH. This study intended to investigate the regulatory mechanism of circRNA on SIRT1 expression in CH. CH model was established by angiotensin II (Ang II) fusion or transverse aortic constriction (TAC) surgery and Ang II treatment on hiPSC-CMs and H9c2 cells in vitro. Our results showed that circ-SIRT1 (hsa_circ_0093884) expression was downregulated in Ang II-treated hiPSC-CMs, and confirmed that its conserved mouse homolog circ-Sirt1 (mmu_circ_0002354) was expressed at low levels in Ang II-treated H9c2 cells and TAC-induced mice model. Functionally, circ-SIRT1/circ-Sirt1 attenuated Ang II-induced CH and induced autophagy in hiPSC-CMs and H9c2 cardiomyocytes. Mechanistically, circ-SIRT1 could upregulate its host gene SIRT1 at the post-transcriptional level by sponging miR-3681-3p/miR-5195-3p and stabilized SIRT1 protein at the post-translational level by recruiting USP22 to induce deubiquitination on SIRT1 protein. Further, SIRT1 knockdown could rescue the effect of circ-SIRT1 upregulation on Ang II-induced CH and autophagy in vitro and in vivo. In conclusion, we first uncovered that circ-SIRT1 restrains CH via activating SIRT1 to promote autophagy, indicating circ-SIRT1 as a promising target to alleviate CH.

## Introduction

Cardiac hypertrophy (CH), featuring enlarged cardiomyocytes as well as heart mass, is generally depicted as a significant compensatory mechanism of the heart in response to diverse physiological and pathological overloads, and it helps sustain cardiac function in its original stage [1, 2]. Unfortunately, continuous CH is related to dysfunction and cardiac remodeling, which ultimately leads to lower compliance, a higher risk of congestive heart failure, and sudden death, so CH has a high rate of death all over the world [3–5]. Pathological CH is a common risk factor for heart failure and multiple factors contributed to CH, including aging and neurohumoral activation (e.g., Ang II) [6]. Anti-aging approaches, such as the restriction of caloric, can benefit cardiac functions in rodents, monkeys as well as human beings [7–9]. Although researchers are working effortlessly to study CH pathology and have uncovered that multiple pathological stimuli (i.e., specific peptide hormones and growth factors) are implicated in CH regulation, it remains difficult to fully explain the complicated molecular mechanisms in CH [10]. Thus, to improve the quality of CH treatment and prevent ultimate heart failure, it is of considerable significance to identify potent therapeutic targets that may modulate CH.

Over the past decade, RNA molecules have been revealed to participate in the regulation of various diseases, including CH [11, 12]. Commonly, mRNAs are involved in the biological process of cancer progression by directly coding functional proteins [13]. SIRT1, a kind of mRNA with protein-coding potential, has been reported to promote autophagy by de-acetylation of multiple autophagy-related genes such as Beclin1 and ATG9A as reported by previous studies [14, 15]. Interestingly, it is acknowledged that promoting autophagy can attenuate CH [16, 17], and accumulating evidence supports that SIRT1 has an inhibitive effect on CH [18–20]. However, the upstream mechanism of regulating SIRT1 expression in CH remains largely unknown.

Most mammalian genomes can be transcribed into noncoding RNAs (ncRNAs) [21]. Traditionally, ncRNAs are predominantly categorized into two subtypes depending on size: long noncoding RNAs (lncRNAs) with over 200 nucleotides in length, and small noncoding RNAs (microRNAs [miRNAs] included) featured with 20–23 nucleotide small RNAs [22]. Emerging as a new star in molecular researches, circular RNAs (circRNAs) are a group of ncRNAs characterized by a closed continuous loop structure, in which the 3’- and 5’-ends are joined together by back splicing [23, 24]. In recent years, circRNAs have also been recognized as competing endogenous RNAs (ceRNAs) which can sponge miRNAs by complementary base pairing. The regulation of circRNAs on SIRT1 has been revealed in different types of human tumors and diseases. For example, hsa_circ_0001946 propels cell growth in lung adenocarcinoma via the serving as a ceRNA regulating miR-135a-5p/SIRT1 axis [25]. Moreover, hsa_circ_0076248 enhances the tumorigenesis of glioma by acting as a miRNA sponge for miR-181a to regulate SIRT1 expression [26]. Circ-SIRT1 (hsa_circ_0093887), derived from the circularization of exon 2 to exon-7 of the SIRT1 gene, has been reported to promote SIRT1 expression by sponging miR-132/212 in vascular smooth muscle cells [27]. Through preliminary experiments, we discovered that circ-SIRT1 was downregulated by Ang II in cardiomyocytes. However, the relation between circ-SIRT1 and SIRT1 in CH has never been studied.

Thus, this study aimed to uncover the regulation of circ-SIRT1 on SIRT1 and the function of circ-SIRT1/SIRT1 axis in CH. We established in vitro and in vivo CH models and applied a string of functional assays and molecular mechanism experiments to probe into the potential upstream regulation of SIRT1 including circ-SIRT1 (hsa_circ_0093884) and its conserved mouse homolog circ-Sirt1 (mmu_circ_0002354) in CH, attempting to identify circ-SIRT1 as a novel target for the improvement of CH treatment.

## Materials and methods

### Cell culture and treatment

Human-induced pluripotent stem cell-derived cardiomyocytes (hiPSC-CMs) were available from Beijing Cellapy Biotechnology Co., Ltd. (Beijing, China), and the rat heart-derived H9c2 cardiomyocytes were available from the Chinese Academy of Sciences (Shanghai, China). Both cardiomyocytes were grown in DMEM (Dulbecco’s Modified Eagle Medium; Invitrogen, Carlsbad, CA, USA) under 5% CO_2_ at 37 °C. In all, 10% FBS (fetal bovine serum; HyClone, South Logan, UT, USA) and 1% antibiotics acted as the medium supplements. The in vitro CH cell model was established in both cardiomyocytes with the 24 h of treatment of 150 nM of Ang II (Sigma-Aldrich, St. Louis, MO, USA) or 50 μM of isoproterenol (ISO; GuideChem, China) for 24 h. In total, 3 U/μg of RNase R was procured from Epicentre Technologies (Madison, WI, USA). The cycloheximide (CHX; 40 μg/mL) was also acquired from Sigma-Aldrich (St. Louis, MO, USA). The 3-methyladenine (3-MA; an autophagy inhibitor) was obtained from Abcam (Cambridge, MA, USA).

### Plasmid transfection

The designed shRNAs and NC-shRNAs (Genepharma Company, Shanghai, China) were applied for silencing circ-SIRT1 (or circ-Sirt1), USP22, and SIRT1. Besides, miR-3681-3p mimics/inhibitor and NC mimics/inhibitor as well as the pcDNA3.1(+)/circ-SIRT1 (circ-SIRT1-oe) or pcDNA3.1(+)/circ-Sirt1 (circ-Sirt1-oe) or empty pcDNA3.1(+) CircRNA Mini Vector (pcDNA3.1(+)), were all procured from Genepharma Company. All plasmids were transfected into hiPSC-CMs and H9c2 cardiomyocytes for 48 h employing the Lipofectamine 2000 (Invitrogen, Carlsbad, CA, USA). Three independent bio-repeats were conducted in the experiment and each bio-repeat contains three technical replicates.

### Animal study

To establish the CH mouse model, male C57BL/6 mice (*n* = 12), aging 8-week-old and weighing 20–25 g, were commercially acquired from Beijing Vital River Laboratory Animal Technology Co., Ltd. (Beijing, China). The animal-related protocol was approved by the Animal Care and Use Committee of the First Hospital of China Medical University. The CH mouse model was established via Transverse Aortic Constriction (TAC) surgery (*N* = 6). After anesthesia with intraperitoneal ketamine and xylazine, the transverse thoracic aorta was dissected from the mouse model. Mice were then placed on a ventilator for 1 week to recover the mouse model. The pressure gradient in the TAC group was examined by echocardiography. Mice of the sham group (*N* = 6) were subjected to the same experimental procedures as the TAC group, except the aorta seam. Besides, we constructed Ang II-treated in vivo model by chronic subcutaneous infusion of Ang II into mice (*N* = 6), saline as control (*N* = 6). To further analyze the effects of circ-Sirt1 on Ang II-treated in vivo model, adenovirus (Biocobio, Tianjin, China) of circ-Sirt1 or pcDNA3.1(+) was injected into the mice left ventricle myocardium 7 days before TAC surgery.

### Enzyme-linked immunosorbent assay (ELISA)

Serum was diluted using PBS, cardiac tissues with the same weight in the sham and TAC groups were prepared via homogenization in PBS. Ang II concentrations in serum were measured via ELISA kit (R&D Systems, Inc., Minneapolis, MN, USA) following the supplier’s protocols. All samples were analyzed via two bio-repeats and two technical replicates.

### Immunofluorescence staining for cell surface area measurement

Mice heart cells, cardiomyocytes (hiPSC-CMs and H9c2) were seeded in 96-well plates and then washed twice with phosphate-buffered saline (PBS). Subsequently, cardiomyocytes were fixed by 4% formaldehyde for 20 min, permeated by 0.1% Triton X-100, and then incubated with α-actinin antibody (Cat #: 6487; 1/100, Cell Signaling Technology, Danvers, MA, USA) at 4 °C overnight. The secondary antibody (Cat #: 4410; 1/1000, Cell Signaling Technology) was used to culture cells at room temperature for 1 h. After DAPI staining, immunofluorescence was observed under a fluorescence microscope (Olympus Corp., Tokyo, Japan) for the estimation of cell surface area. In all, 50 independent cells were analyzed per sample. The researchers performing the analysis were blinded to the experimental groups. Three independent bio-repeats were conducted in the experiment and each bio-repeat contains three technical replicates. The results were analyzed with Image-Pro Plus Data Analysis software.

### RNA extraction and RT-qPCR

TRIZOL reagent (Invitrogen, Carlsbad, CA, USA) was used for the extraction of total RNA from cardiomyocytes. Then, the reverse transcription was accomplished as per the supplier’s instructions (Invitrogen, Carlsbad, CA, USA). To examine gene expression, RT-qPCR was carried out on ABI 7500 Fast Real-Time PCR System (Applied Biosystems, Foster City, CA, USA). Data were processed by 2^-∆∆Ct^ method and standardized to U6 or GAPDH (glyceraldehyde-3-phosphate dehydrogenase). Three independent bio-repeats were conducted in the experiment and each bio-repeat contains three technical replicates.

### Western blot

The cultured cardiomyocytes were lysed in RIPA lysis buffer, then separated on 12% SDS-PAGE (sodium dodecyl sulfate-polyacrylamide gel electrophoresis) and transferred to PVDF membranes. After being blocked in 5% skim milk, primary antibodies against GAPDH (Cat. #: ab8245; 1/5000, Abcam, Cambridge, UK; control) and ANF (atrial natriuretic factor; Cat. #: ab225844, 1/1000, Abcam), BNP (brain natriuretic peptide; Cat. #: ab92500, 1/10,000 Abcam), β-MHC (β-myosin heavy chain; Cat. #: ab170867, 1/3000, Abcam), p62 (Cat. #: ab109012, 1/30,000, Abcam), LAMP1 (lysosomal associated membrane protein 1; Cat. #: ab108597, 1/5000, Abcam), SIRT1 (Cat. #: ab32441, 1/20,000, Abcam) and USP22 (ubiquitin-specific peptidase 22; Cat. #: ab195289, 1/2000, Abcam) were used. Anti-LC3 (light chain 3) antibody (Cat. #: 14600-1-AP, 1/1500, Proteintech, Rosemont, IL, USA) was procured from Sigma-Aldrich (St. Louis., MO, USA). To be noted, there are two forms of LC3 in various cells, called LC3-I and -II; LC3-I is cytosolic, whereas LC3-II is membrane-bound [28]. After overnight incubation, the HRP-tagged secondary antibodies (Cat. #: ab6728; 1/5000, Abcam) were added for 2 h at room temperature. All protein bands were examined by the ECL detection system (Pierce, Rockford, IL, USA). Western blot results of proteins of interest in the cardiomyocytes were quantified with the help of ImageJ software and statistical analysis was conducted via SPSS version 13.0. Three independent bio-repeats were conducted in the experiment and each bio-repeat contains three technical replicates.

### Subcellular fractionation

In total, 1 × 10^6^ hiPSC-CMs and H9c2 cardiomyocytes were washed in pre-cooled PBS twice for 5 min of centrifugation at 500×*g* at 4 °C. Cytoplasmic and nuclear fractions were individually extracted using PARIS™ Kit (Invitrogen, Carlsbad, CA, USA) following the manufacturer’s direction. Expression levels of circ-SIRT1 (or circ-Sirt1) in both fractions were assayed by RT-qPCR, with GAPDH and U6 as controls for cell cytoplasm and cell nucleus, respectively. Three independent bio-repeats were conducted in the experiment and each bio-repeat contains three technical replicates.

### Fluorescence in situ hybridization (FISH)

H9c2 and hiPSC-CMs cardiomyocytes were fixed and processed with pepsin, then dehydrated in ethanol. Cells were incubated in hybridization buffer with 40 nM of circ-SIRT1 (or circ-Sirt1)-FISH probe (Ribobio, Guangzhou, China). Following nuclear detection with DAPI solution, cells were detected visually by fluorescence microscope (Olympus Corp., Tokyo, Japan). Three independent bio-repeats were conducted in the experiment and each bio-repeat contains three technical replicates.

### GFP-mRFP-LC3 adenoviral transfection

H9c2 and hiPSC-CMs cardiomyocytes were planted on the glass-bottomed cell culture dishes and then infected with the adenoviral vectors GFP-mRFP-LC3 (Ad-GFP-mRFP-LC3; HanBio Technology, Shanghai, China) expressing the GFP and mRFP fluorescent proteins for marking and tracking LC3 to monitor autophagic influx. Then, the culture medium was changed with the fresh medium, and cells were observed by confocal laser scanning microscope (Zeiss, Dublin, CA, USA) after 24 h to analyze autophagy flux and the number of green, red (autolysosomes), and yellow dots (autophagosomes), which were quantified with the application of Image Plus Pro Software. More than ten cells in three independent bio-repeats were analyzed randomly in the experiment and each bio-repeat contains three technical replicates.

### RNA pull-down assays

For RNA-RNA pull-down assays, the cardiomyocytes were subjected to ice-cold PBS and lysis buffer and then incubated with circ-SIRT1 biotin probes (TCTGAAGAGCTCTGTGACCC-biotin) or NC-biotin probes (AGACTTCTCGACTGTGACCC-biotin) at room temperature for 2 h. After the addition of streptavidin magnetic beads (Invitrogen, Carlsbad, CA, USA), the biotin-coupled circ-SIRT1 complex was incubated for another 4 h at 4 °C, followed by washing with RIP washing buffer. Subsequently, the binding miRNAs were extracted from the pulldowns using TRIzol and detected using qRT-PCR. Enrichment values of miRNA (miR-3681-3p, miR-4766-5p, miR-889-3p, or miR-5195-3p) were compared to NC-biotin probe control.

To verify the interaction between miR-3681-3p/miR-5195-3p and circ-SIRT1 and SIRT1, biotinylated miR-3681-3p or miR-5195-3p (Bio-miR-3681-3p: acacagugcuucauccacuacu-biotin; Bio-miR-5195-3p: auccaguucucugagggggcu-biotin) was incubated with streptavidin magnetic beads. Then, circ-SIRT1 and SIRT1 in the pulldowns were isolated using TRIzol and subjected to qRT-PCR analysis. Enrichment values of circ-SIRT1/SIRT1 were relative to Bio-NC control, and the data were mean ± standard variation (SD) of three replicates. For RNA-protein pull-down assays, Pierce Magnetic RNA-Protein Pull-Down Kit was acquired from Thermo Fisher (Waltham, MA) for RNA-protein pull-down assay in cardiomyocytes. The protein extracts were prepared and set as three groups, including Input, circ-SIRT1 biotin probe, and circ-SIRT1 no-biotin probe. The protein extracts were mixed with the biotin-tagged circ-SIRT1 probes. The magnetic beads were added for 1 h. Following RNA-protein pull-down assay, the circ-SIRT1 interacting proteins were subjected to SDS-PAGE and silver staining, followed by mass spectrometry analysis (CapitalBio Technology, Beijing, China) of the specific band. Besides, the enriched USP22 was detected by western blot. Three independent bio-repeats were conducted in the experiment and each bio-repeat contains three technical replicates.

### RNA immunoprecipitation (RIP)

Magna RIP™ RNA-Binding Protein Immunoprecipitation Kit was available for RIP assay in 1 × 10^7^ cardiomyocytes as required by the supplier (Millipore, Bedford, MA, USA). Cell lysates were incubated with RIP buffer adding the magnetic beads and specific antibodies to human Ago2 (Cat. #: 2897; 1/50, Cell Signaling Technology) and USP22 (Cat. #: ab195289; 1/40, Abcam). Normal mouse IgG antibody (Cat. #: 3420; 1/20, Cell Signaling Technology) was used in the control group. Three independent bio-repeats were conducted in the experiment and each bio-repeat contains three technical replicates.

### Co-immunoprecipitation (CoIP)

The cardiomyocytes were harvested by centrifugation at 400×*g* for 3 min. The processed cardiomyocytes were re-suspended in IP lysis buffer and subsequently left on ice for 15 min. After that, the cardiomyocytes were sonicated for 2 × 10 s and placed directly on ice, followed by centrifugation at 10,000×*g* for 10 min at 4 °C. Afterward, the supernatant was transferred to a fresh cold Eppendorf tube. Bradford assay was applied to determine the protein concentration. The lysate was incubated with the specific primary antibodies against myc (Cat. #: 2276; 1/1000, Cell Signaling Technology), Flag (Cat. #: 14793; 1/50, Cell Signaling Technology), SIRT1 (Cat. #: 8469; 1/100, Cell Signaling Technology), USP22 (Cat. #: ab195289, 1/40, Abcam), c-Myc (Cat. #: ab32072; 5 µg/ml, Abcam), Ub (ubiquitin; Cat. #: 62802; 1/100, Cell Signaling Technology), or IgG antibody (Cat. #: 3420; 1/20, Cell Signaling Technology) plus A/G sepharose beads or A/G sepharose beads only on a rotator overnight at 4 °C. Then, cell samples were washed in IP lysis buffer and boiled in 4× SDS-loading buffer for western blot analysis. Three independent bio-repeats were conducted in the experiment and each bio-repeat contains three technical replicates.

### Luciferase reporter assay

The miR-3681-3p target sequences (wild-type or mutated) within the SIRT1 fragment were inserted in pmirGLO luciferase vector (Promega, Madison, WI), named as SIRT1-WT and SIRT1-Mut vectors. After co-transfection with NC mimics, miR-3681-3p mimics or miR-3681-3p mimics + circ-SIRT1-oe, cardiomyocytes were assayed with Luciferase Reporter Assay System (Promega). In addition, cardiomyocytes were co-transfected with circ-SIRT1-oe or NC-pcDNA3.1(+), and pGL3 vector containing SIRT1 promoter or pmirGLO vector covering SIRT1 3’UTR for luciferase assay. Three independent bio-repeats were conducted in the experiment and each bio-repeat contains three technical replicates.

### Statistical analyses

Data were analyzed by SPSS version 13.0 (SPSS, Chicago, IL) and displayed as the mean ± SD. Experimental results were analyzed by one-way or two-way analysis of variance (ANOVA) or Student’s *t* test, with two-tailed *P* value below 0.05 as a significant level.

## Results

### Circ-SIRT1 and its homologous circ-Sirt1 are low-expressed in the CH model in vitro and in vivo

**Fig. 1:**
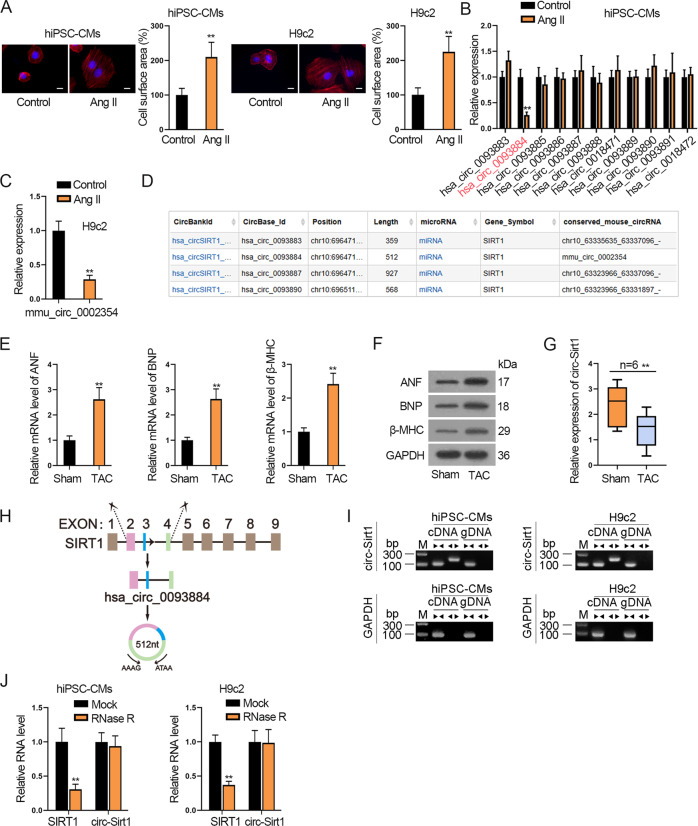
Circ-SIRT1 and its homologous circ-Sirt1 are low-expressed in the CH model in vitro and in vivo. **A** The surface area of hiPSC-CMs and H9c2 cells treated without (control) or Ang II was observed by immunofluorescence staining of α-actinin (red). Scale bar, 10 μm. *N* = 3. **B** RT-qPCR was applied to analyze the expressions of circRNAs associated with SIRT1 gene in Ang II-treated hiPSC-CMs. *N* = 3. **C** RT-qPCR analysis of mmu_circ_0002354 (circ-Sirt1) expression in Ang II-treated H9c2 cells versus non-treated H9c2 cells. *N* = 3. **D** According to the searching results in bioinformatics website circBank, mmu_circ_0002354 (circ-Sirt1) in conserved human homolog is hsa_circ_0093884 (circ-SIRT1). **E**, **F** The expression of CH markers (ANF, BNP, and β-MHC) in the TAC group (*N* = 6) and sham group (*N* = 6) was examined via RT-qPCR and western blot analyses. **G** RT-qPCR detection of circ-Sirt1 expression in the hearts of mice subjected to TAC surgery (TAC group, *N* = 6) and sham operation (sham group, *N* = 6). **H** Schematic illustration displayed the circularization of SIRT1 exon 2–4 to form hsa_circ_0093884 (circ-SIRT1). **I** Nucleic acid electrophoresis uncovered that divergent primers could produce the circular isoform of SIRT1 (or Sirt1) with cDNA but not with gDNA in hiPSC-CMs (or H9c2 cells). GAPDH was an internal control. *N* = 3. **J** The abundance of circ-SIRT1/circ-Sirt1 or linear RNA SIRT1/Sirt1 analyzed via RT-qPCR after RNase R treatment in hiPSC-CMs (or H9c2 cells). *N* = 3. ^**^P < 0.01 was assessed by Student’s *t* test.

First, we treated human hiPSC-CMs and mouse H9c2 cells with Ang II, a known method to induce CH [29, 30], to establish in vitro CH model. After treatment, the characteristics of CH were monitored by measuring cell surface area and sarcomere number. The results showed that the surface area of hiPSC-CMs and H9c2 cells was enlarged (Fig. 1A), and the ratio of well-organized hiPSC-CMs and H9c2 cells increased significantly after Ang II treatment (Supplementary Fig. 1A). So we confirmed that the CH cell model was successfully built by Ang II treatment.

Then, we searched circBase (http://www.circbase.org/) to find out circRNAs related to SIRT1. Consequently, 11 homo sapiens circRNAs and 1 mouse homolog circRNA were respectively associated with SIRT1 (human) and Sirt1 (mouse) gene (Supplementary Table 2**)**. After Ang II treatment, only the expression of hsa_circ_0093884 was markedly downregulated in hiPSC-CMs (Fig. 1B). Similarly, mmu_circ_0002354 was low-expressed in Ang II-treated H9c2 cells (Fig. 1C). Interestingly, searching circBank (http://www.circbank.cn/), we discovered that mmu_circ_0002354 happened to be a conserved mouse homolog for hsa_circ_0093884 (Fig. 1D). Thus, we speculated that hsa_circ_0093884 and its homologous mmu_circ_0002354 were involved in CH. So we named hsa_circ_0093884 as circ-SIRT1 and mmu_circ_0002354 as circ-Sirt1 for further assays.

Then, to detect circ-Sirt1 level in CH in vivo, mouse CH model was built by TAC surgery. First, RT-qPCR and western blot analyses confirmed the upregulation of CH markers (ANF, BNP, and β-MHC) in TAC group (Fig. 1E, F and Supplementary Fig. 1B). Also, Ang II serum level was significantly high in the TAC group than sham group (Supplementary Fig. 1C). These data confirmed the establishment of CH model in vivo. Then, we verified that circ-Sirt1 expression was lower in the TAC group than that in the sham group (Fig. 1G), indicating that circ-Sirt1 participated in CH.

Subsequently, the genomic location and the splicing pattern of circ-SIRT1 are illustrated in Fig. 1H. Nucleic acid electrophoresis uncovered that divergent primers could produce the circular isoform of SIRT1 (or Sirt1) with cDNA but not with gDNA, whereas convergent primers could amplify the linear isoform of SIRT1 (or Sirt1) from both cDNA and gDNA in hiPSC-CMs (or H9c2 cells) (Fig. 1I). After RNase R treatment, circ-SIRT1 (or circ-Sirt1) presented higher stability in contrast to linear SIRT1 (or Sirt1) (Fig. 1J).

Taken together, circ-SIRT1 and circ-Sirt1 were characterized with closed-loop structure and expressed at low levels in Ang II-treated cell models. Besides, circ-Sirt1 was low-expressed in the TAC mice model.

### Circ-SIRT1 and homologous circ-Sirt1 inhibit CH and contribute to autophagy in hiPSC-MCs and H9c2 cells

**Fig. 2:**
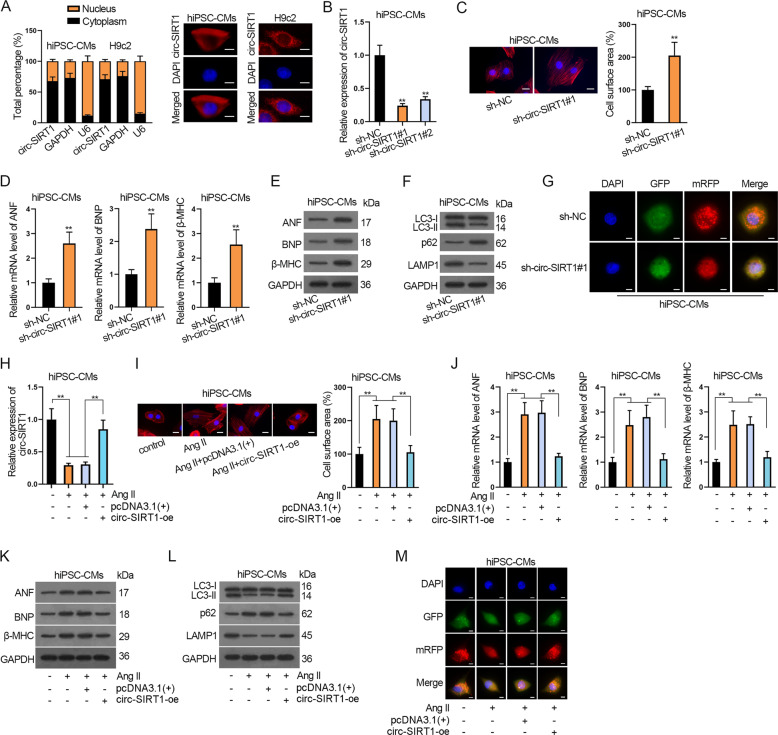
Circ-SIRT1 inhibits CH and contributes to autophagy in hiPSC-MCs. **A** The subcellular distribution of SIRT1 (or Sirt1) was detected via subcellular fractionation and FISH assays. Scale bar, 10 μm. *N* = 3. **B** RT-qPCR analysis of circ-SIRT1 knockdown by sh-circ-SIRT#1/2 in hiPSC-CMs is shown. *N* = 3. **C** Analysis of the effects of circ-SIRT1 deficiency on cell surface area via immunofluorescence staining. Scale bar, 10 μm. *N* = 3. The percentage of cell surface area of the sh-circ-SIRT1#1 group to that of the sh-NC group was evaluated. **D**, **E** The expressions of CH markers in hiPSC-CMs transfected with sh-NC or sh-circ-SIRT1#1 were examined via RT-qPCR and western blot analyses. **F** Western blot analysis of levels of autophagy-related proteins (LC3-II/LC3-I, p62, and LAMP1) in hiPSC-CMs transfected with sh-NC or sh-circ-SIRT1#1. *N* = 3. **G** The hiPSC-CMs were transfected with Ad-GFP-mRFP-LC3 for 48 h and sh-NC or sh-circ-SIRT1#1. The change of both GFP fluorescence (green) and mRFP (red) fluorescence was observed by confocal microscopy. Scale bar, 10 μm. *N* = 3. **H** Circ-SIRT1 expression in hiPSC-CMs treated without or with Ang II and transfected with pcDNA3.1(+) or pcDNA3.1(+)/circ-SIRT1 (circ-SIRT1-oe) was examined via RT-qPCR. *N* = 3. **I** Cell surface area of hiPCS-CMs treated without or with Ang II and transfected with pcDNA3.1(+) or pcDNA3.1(+)/circ-SIRT1 (circ-SIRT1-oe) was analyzed by immunofluorescence staining. Scale bar, 10 μm. *N* = 3. **J**, **K** The expression of CH markers in hiPSC-CMs of the above-mentioned groups was examined via RT-qPCR and western blot analyses. *N* = 3. **L**, **M** The effect of upregulated circ-SIRT1 on autophagy influx in hiPSC-CMs treated without or with Ang II and transfected with pcDNA3.1(+) or pcDNA3.1(+)/circ-SIRT1 (circ-SIRT1-oe) was analyzed via western blot analysis of autophagy-related proteins and immunofluorescence staining analysis of Ad-GFP-mRFP-LC3. Representative images of GFP dots (green), mRFP dots (red), and their merged images are shown. Scale bar, 10 μm. *N* = 3. ^**^P < 0.01 was assessed by Student’s *t* test for comparison between groups, one-way ANOVA and Tukey for multiple groups.

Thereafter, we explored the effect of circ-SIRT1 (or circ-Sirt1) on CH. First, subcellular fractionation and FISH detection depicted that circ-SIRT1 and circ-Sirt1 were mainly distributed in the cytoplasm of hiPSC-CMs and H9c2 cells, respectively (Fig. 2A). As depicted by RT-qPCR data, sh-circ-SIRT1#1/2 and sh-circ-Sirt1#1/2 decreased circ-SIRT1 level in hiPSC-CMs and circ-Sirt1 level in H9c2 cells (Fig. 2B and Supplementary Fig. 1D). Also, FISH images depicted an efficiency circ-SIRT1 or circ-Sirt1 knockdown by sh-circ-SIRT1#1/2 or sh-circ-Sirt1#1/2 (Supplementary Fig. 1E). Since sh-circ-SIRT1#1 and sh-circ-Sirt1#1 presented better knockdown efficiency, we used these shRNAs for functional experiments.

Next, CH was determined by measuring cell surface area, sarcomere organization, and CH markers. Consequently, circ-SIRT1 silencing observably enlarged cell surface area and increased the ratio of well-organized cardiomyocytes of hiPSC-CMs (Fig. 2C and Supplementary Fig. 1F). In addition, knockdown of circ-SIRT1 notably elevated mRNA and protein levels of ANF, BNP, and β-MHC, indicating that circ-SIRT1 deficiency was conducive to CH formation (Fig. 2D, E and Supplementary Fig. 1G).

Autophagy is known to be crucial to CH development [16, 31], and SIRT1 (or Sirt1), the associated gene for circ-SIRT1 (or circ-Sirt1), has been proved to regulate autophagy in CH [14, 15]. Therefore, we evaluated autophagic flux in Ang II-treated hiPSC-CMs. Western blot showed that protein levels of LC3-II/LC3-I and LAMP1 were significantly decreased by circ-SIRT1 depletion, whereas that of p62, a polyubiquitin-binding protein known to be sequestered and degraded during autophagy [32], was markedly elevated (Fig. 2F and Supplementary Fig. 1H), suggesting the inhibitive effect of circ-SIRT1 downregulation on autophagy. Besides, the autophagic influx was monitored via using Ad-GFP-mRFP-LC3 expressing the GFP and mRFP fluorescent proteins for marking and tracking LC3. As the GFP fluorescent protein is sensitive to acidity, GFP fluorescence stain was quenched when lysosome-autophagosome fusion happens, only detecting red dots (mRFP). In other words, the decrease of GFP indicates the lysosome fuses with autophagosome to form autolysosome. Our results showed the numbers of GFP and mRFP-positive dots per cell were both significantly decreased after the downregulation of circ-SIRT1, so fewer red dots than yellow dots were seen in the merged images, and both autophagosomes and autolysosomes decreased under circ-SIRT1 knockdown (Fig. 2G and Supplementary Fig. 1I), indicating that both autolysosome formation and LC3 expression were impeded by circ-SIRT1 knockdown in hiPSC-CMs. Thus, circ-SIRT1 knockdown restrained autophagic flux.

Meanwhile, the effect of circ-SIRT1 upregulation on CH and autophagy in hiPSC-CMs was analyzed. To begin with, circ-SIRT1-oe significantly elevated circ-SIRT1 expression in Ang II-infused hiPSC-CMs (Fig. 2H). Unsurprisingly, circ-SIRT1 upregulation reversed the enlargement of cell surface area and the increase of well-organized cardiomyocytes ratio of hiPSC-CMs treated with Ang II (Fig. 2I and Supplementary Fig. 1J). Likewise, the upregulated circ-SIRT1 could reverse the inductive effect of Ang II on the mRNA and protein levels of CH markers (Fig. 2J, K and Supplementary Fig. 1K). Further, western blot analysis of autophagy-related proteins and immunofluorescence staining analysis of Ad-GFP-mRFP-LC3 were performed. The results uncovered that elevating circ-SIRT1 expression offset the effect of Ang II on decreasing LC3-II/LC3-I and LAMP1 levels and increasing p62 level (Fig. 2L and Supplementary Fig. 1L). Ang II decreased the percentage of positive GFP dots and mRFP dots relative to total cells and the number of autophagosomes and autolysosomes per cell, and circ-SIRT1-oe reversed such effect (Fig. 2M and Supplementary Fig. 1M). Taken together, circ-SIRT1 inhibits CH formation and promotes autophagy.

Similarly, we conducted same experiments to explore the effect of circ-Sirt1 in H9c2 cells. Results showed that circ-Sirt1 knockdown increased the ratio of well-organized cells and levels of ANF, BNP, β-MHC, and inhibited autophagy in H9c2 cells (Supplementary Fig. 2A–E). Also, Ang II-inhibited circ-Sirt1 level was restored by circ-Sirt1 overexpression in H9c2 cells (Supplementary Fig. 2F). Then, we verified that Ang II promoted CH development and impeded autophagy in H9c2 cells, and such effects were offset by overexpressing circ-Sirt1 (Supplementary Fig. 2G–K). To sum up, circ-SIRT1 and circ-Sirt1 not only impair CH formation but also promote autophagy.

### Circ-SIRT1 suppresses CH by promoting autophagy in hiPSC-CMs

**Fig. 3:**
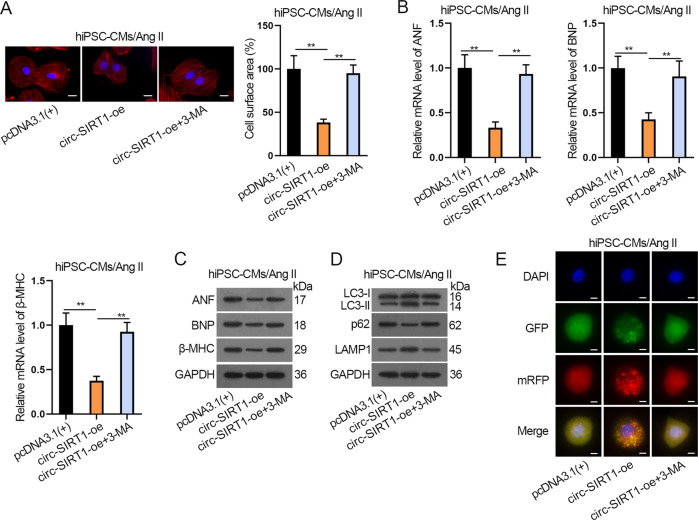
Circ-SIRT1 suppresses CH by promoting autophagy in hiPSC-CMs. **A** Analysis of the cell surface area via immunofluorescence staining after Ang II-infused hiPSC-CMs transfected with pcDNA3.1(+) or circ-SIRT1-oe or circ-SIRT1-oe plus treatment with 3-MA (autophagy inhibitor). Scale bar, 10 μm. *N* = 3. **B**, **C** The expressions of CH markers in Ang II-infused hiPSC-CMs of the above-mentioned groups were determined by RT-qPCR and western blot analyses. *N* = 3. **D**, **E** Autophagy was measured via western blot analysis of autophagy-related proteins and immunofluorescence staining analysis of Ad-GFP-mRFP-LC3 in Ang II-infused hiPSC-CMs of the above-mentioned groups. Representative images of fluorescent GFP dots (green), mRFP dots (red), and their merged images were shown. Scale bar, 10 μm. *N* = 3. ^**^P < 0.01 was assessed by one-way ANOVA and Tukey.

Next, we conducted a series of rescue assays to verify whether circ-SIRT1 modulates CH by regulating autophagy. In the period, we used 3-methyladenine (3-MA) to interfere with autophagy. As illustrated in Fig. 3A and Supplementary Fig. 3A, the addition of 3-MA completely restored the restraining effect of circ-SIRT1 upregulation on cell surface area and the ratio of well-organized cardiomyocytes of Ang II-treated hiPSC-CMs. Unsurprisingly, the suppressive influence of circ-SIRT1 overexpression on the expression of ANF, BNP, and β-MHC was entirely rescued by adding 3-MA to Ang II-treated hiPSC-CMs (Fig. 3B, C and Supplementary Fig. 3B). Thereby, it was suggested that circ-SIRT1 might repress CH by promoting autophagy. For further verification, we analyzed the levels of autophagy-related proteins. The results showed that 3-MA treatment reversed the promoting effect of circ-SIRT1 overexpression on LC3-II/LC3-I and LAMP1 protein levels and inhibitory effect of circ-SIRT1 overexpression on p62 protein in Ang II-treated hiPSC-CMs (Fig. 3D and Supplementary Fig. 3C). Also, we evaluated the extent of autophagic flux. The promoting effect of circ-SIRT1 overexpression on the numbers of GFP and mRFP-positive dots per cell were both reversed by 3-MA treatment; fewer red dots than yellow dots were seen in the merged images of circ-SIRT-oe+3-MA group compared with circ-SIRT1-oe group in Ang II-treated hiPSC-CMs, indicating significantly decreased autolysosome formation compared with autophagosomes (Fig. 3E and Supplementary Fig. 3D). Taken together, the results above elucidated that the addition of 3-MA could completely reverse the facilitating effect of upregulated circ-SIRT1 on autophagy in Ang II-treated hiPSC-CMs.

Therefore, circ-SIRT1 impedes CH by promoting autophagy.

### Circ-SIRT1 recruits USP22 to regulate the deubiquitination and stabilization of SIRT1 in hiPSC-CMs

**Fig. 4:**
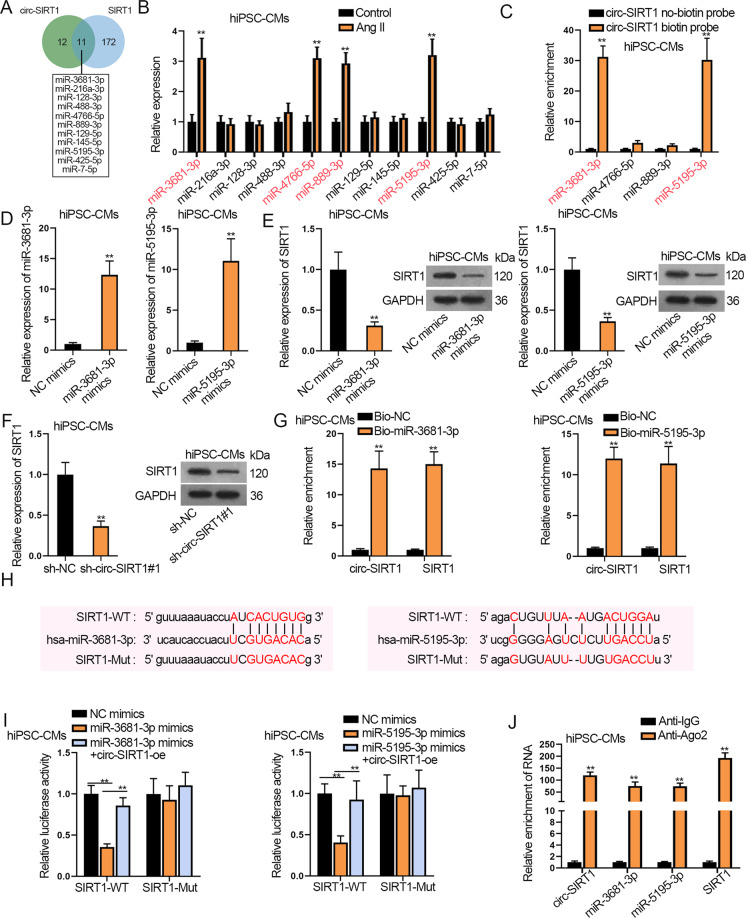
Circ-SIRT1 upregulates SIRT1 expression by sequestering miR-3681-3p/miR-5195-3p in hiPSC-CMs. **A** In total, 11 miRNAs were predicted by starBase to possess the binding capacity with circ-SIRT1 (or SIRT1). **B** The expression levels of candidate miRNAs in Ang II-infused hiPSC-CMs versus control hiPSC-CMs were detected via RT-qPCR. *N* = 3. **C** RT-qPCR results of the enrichment of four miRNAs in pulldown products of circ-SIRT1 no-biotin probe or circ-SIRT1 biotin probe. Enrichment values of miRNAs were normalized to NC-biotin probe control. *N* = 3. **D** RT-qPCR analysis of the upregulation of miR-3681-3p and miR-5195-3p in hiPSC-CMs with the transfection of miR-3681-3p mimics and miR-5195-3p mimics, respectively. *N* = 3. **E**, **F** Analysis of SIRT1 expression via RT-qPCR and western blot assays in hiPSC-CMs transfected with miR-3681-3p/miR-5195-3p mimics versus NC mimics or transfected with sh-circ-SIRT1#1 versus sh-NC. *N* = 3. **G** RT-qPCR analysis of the enrichment of circ-SIRT1 and SIRT1 in the pulldown products of Bio-miR-3681-3p/miR-5195-3p and Bio-NC. *N* = 3. Enrichment values of circ-SIRT1/SIRT1 were normalized to Bio-NC control. **H** The binding sites for miR-3681-3p/miR-5195-3p in SIRT1 were predicted by starBase and the sites were mutated by substitution with complementary sites (marked in red). **I** Luciferase activities of SIRT1-WT (with wild-type miR-3681-3p/miR-5195-3p sites) and SIRT-Mut (with mutated miR-3681-3p/miR-5195-3p sites) were evaluated by luciferase reporter assay in hiPSC-CMs transfected with NC mimics, miR-3681-3p/miR-5195-3p mimics or miR-3681-3p/miR-5195-3p mimics+circ-SIRT1-oe. *N* = 3. **J** The enrichment of circ-SIRT1, miR-3681-3p, miR-5195-3p, and SIRT1 in Ago2 RIP products was verified through RIP and RT-qPCR. *N* = 3. ^**^P < 0.01 was assessed by Student’s *t* test for comparison between groups, one-way ANOVA/two-way ANOVA and Tukey for multiple groups.

Then, we tried to explain the mechanism behind the regulation of circ-SIRT1 on SIRT1 in hiPSC-CMs. First, we carried out luciferase reporter assay and observed that circ-SIRT1 upregulation caused no obvious alteration in the luciferase activity of SIRT1 promoter whereas overexpressing HIC2, a known activator for SIRT1 transcription in cardiomyocytes [33], successfully induced the luciferase activity of SIRT1 reporter (Supplementary Fig. 3E, F), but circ-SIRT1 overexpression elevated the luciferase activity of SIRT1 3’UTR (Supplementary Fig. 3G). This suggested the post-transcriptional regulation of circ-SIRT1 on SIRT1. Then, through RNA pull-down assay, we recognized that circ-SIRT1 could not bind with SIRT1 (Supplementary Fig. 3H). Next, circ-SIRT1 was unveiled to be highly enriched in Ago2 RIP precipitates (Supplementary Fig. 3I). Since Ago2 is a core protein in the RNA-induced silencing complex (RISC), the results implied that circ-SIRT1 existed in RISC. Combining with the former finding that circ-SIRT1 was mainly distributed in the cytoplasm, we speculated that circ-SIRT1 might regulate SIRT1 expression by acting as a ceRNA in hiPSC-CMs. Searching starBase (http://starbase.sysu.edu.cn/), we predicted 11 miRNAs possessing the binding capacity with both circ-SIRT1 and SIRT1 (Fig. 4A). Among them, only miR-3681-3p, miR-4766-5p, miR-889-3p, and miR-5195-3p were detected to be apparently overexpressed in Ang II-infused hiPSC-CMs (Fig. 4B). However, only miR-3681-3p and miR-5195-3p were confirmed to be capable of binding with circ-SIRT1 in hiPSC-CMs according to the results of RNA pull-down assay (Fig. 4C). Therefore, we proceeded to examine whether circ-SIRT1 regulated SIRT1 through binding with miR-3681-3p and miR-5195-3p. First, RT-qPCR analysis revealed a favorable upregulation of miR-3681-3p and miR-5195-3p in hiPSC-CMs transfected with miR-3681-3p mimics and miR-5195-3p mimics (Fig. 4D). According to RT-qPCR and western blot analyses, SIRT1 expression was proved to be decreased by the transfection of miR-3681-3p mimics, miR-5195-3p mimics, or sh-circ-SIRT1#1 (Fig. 4E, F and Supplementary Fig. 3J, K). Later on, through RNA pull-down assay, we confirmed that miR-3681-3p and miR-5195-3p could bind with circ-SIRT1 (or SIRT1) in hiPSC-CMs because we observed that circ-SIRT1 (or SIRT1) was highly abundant in Bio-miR-3681-3p and Bio-miR-5195-3p groups (Fig. 4G). In addition, Fig. 4H showed the binding sites for miR-3681-3p/miR-5195-3p on SIRT1, and SIRT1-Mut were designed by substituting the predicted sites with complementary sequences. Then, luciferase reporter assay indicated that upregulation of miR-3681-3p or miR-5195-3p largely weakened the luciferase activity of pmirGLO-SIRT1-WT but had no marked effect on that of pmirGLO-SIRT1-Mut; such effect on pmirGLO-SIRT1-WT was reversed by elevating circ-SIRT1 expression, suggesting the competitive relation between circ-SIRT1 and SIRT1 (Fig. 4I). At last, the RIP assay delineated the significant enrichment of circ-SIRT1, miR-3681-3p, miR-5195-3p, and SIRT1 in the anti-Ago2 group but not in the anti-IgG group, uncovering the coexistence of these RNAs in RISC (Fig. 4J). To conclude, circ-SIRT1 elevates SIRT1 expression by competitively binding with miR-3681-3p/miR-5195-3p in hiPSC-CMs.

### Circ-SIRT1 recruits USP22 to stabilize SIRT1 protein in hiPSC-CMs

**Fig. 5:**
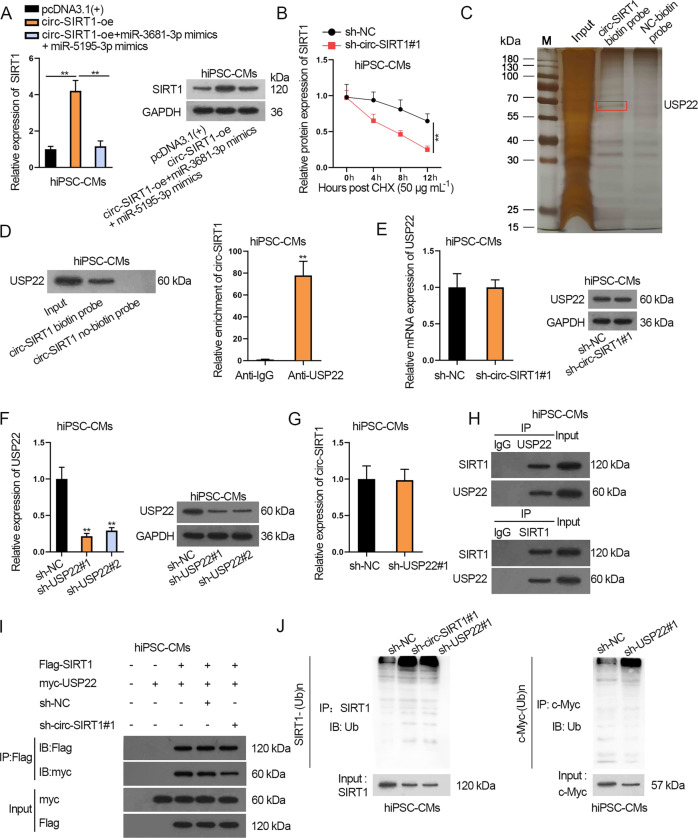
Circ-SIRT1 recruits USP22 to regulate the deubiquitination and stabilization of SIRT1 in hiPSC-CMs. **A** SIRT1 mRNA and protein expression in hiPSC-CMs transfected with pcDNA3.1(+), circ-SIRT1-oe, or circ-SIRT1-oe+miR-3681-3p mimics+miR-5195-3p mimics were analyzed via RT-qPCR and western blot analyses. *N* = 3. **B** SIRT1 protein stability was analyzed by western blot at 0, 4, 8, and 12 h after CHX treatment in hiPSC-CMs. *N* = 3. **C** Silver staining of differential proteins in RNA pull-down products for circ-SIRT1 biotin probe compared with NC-biotin probe. The differential bands were sent to mass spectrometry and USP22 was shown to be significantly enriched in circ-SIRT1 biotin probe group in hiPSC-CMs. *N* = 3. **D** Western blot band of USP22 in RNA pull-down products for circ-SIRT1 biotin probe and circ-SIRT1 no-biotin probe. RT-qPCR results of circ-SIRT1 enrichment in RIP precipitates for anti-IgG and anti-USP22. *N* = 3. **E** RT-qPCR and western blot analyzed the effect of silenced circ-SIRT1 on USP22 expression. *N* = 3. **F** RT-qPCR and western blot analyses of the efficiency of USP22 knockdown by sh-USP22#1/2 in hiPSC-CMs. *N* = 3. **G** RT-qPCR analysis of the effect of silenced USP22 on circ-SIRT1 expression. *N* = 3. **H** Western blot band of SIRT1 and USP22 in the CoIP products for anti-IgG and anti-USP22. *N* = 3. **I** The hiPSC-CMs were transfected with exogenous Flag-tagged SIRT1 and myc-tagged USP22 proteins and sh-NC or sh-circ-SIRT1#1. Western blot band showed the enrichment of myc in the CoIP products in anti-Flag in sh-NC and sh-circ-SIRT1#1 groups. *N* = 3. **J** Analysis of the effect of circ-SIRT1 depletion or USP22 deficiency on SIRT1 ubiquitination is shown, taking c-Myc as a positive control for IP. *N* = 3. ^**^P < 0.01 was assessed by Student’s *t* test for comparison between groups, one-way ANOVA and Tukey for multiple groups.

Interestingly, RT-qPCR and western blot analyses revealed that co-transfection of miR-3681-3p mimics plus miR-5195-3p mimics could only completely restore SIRT1 mRNA level rather than SIRT1 protein level that were reduced by circ-SIRT1 knockdown in hiPSC-CMs (Fig. 5A and Supplementary Fig. 4A). Thus, we speculated that circ-SIRT1 might regulate SIRT1 protein in other ways. CHX chase experiment revealed that circ-SIRT1 depletion accelerated SIRT1 protein degradation in hiPSC-CMs (Fig. 5B). Previous studies have clarified that SIRT1 protein stabilization was related to enzyme-mediated ubiquitination [34, 35]. Therefore, we surmised that circ-SIRT1 might regulate SIRT1 protein stability through certain proteins. The silver staining results of RNA pull-down showed the differential proteins pulled down by circ-SIRT1 biotin probe rather than NC-biotin probe (Fig. 5C). Later, these proteins were subjected to mass spectrometry analysis and results showed that USP22 was prominently enriched in the pulldown products for circ-SIRT1 in hiPSC-CMs (Supplementary Table 3). RNA pulldown and RIP assays also confirmed the strong binding capacity between circ-SIRT1 and USP22 in hiPSC-CMs (Fig. 5D). Importantly, USP22 was reported to stabilize SIRT1 by deubiquitination to promote autophagy in cancer cells and also protect against ischemia–reperfusion injury in cardiomyocytes [36, 37]. Therefore, we speculated that circ-SIRT1 might regulate SIRT1 protein stability via USP22 and proceeded to explore the detailed mechanism. The results of RT-qPCR and western blot showed that circ-SIRT1 knockdown caused no evident changes in USP22 expression (Fig. 5E and Supplementary Fig. 4B). After that, we knocked down USP22 by sh-USP22#1/2 and verified the knockdown efficiency (Fig. 5F and Supplementary Fig. 4C). Likewise, depleting USP22 expression in hiPSC-CMs resulted in no clear changes of circ-SIRT1 expression (Fig. 5G). Taken together, circ-SIRT1 and USP22 cannot regulate each other. Thus, we further investigated whether circ-SIRT1 recruited USP22 to stabilize SIRT1 protein. The CoIP results indicated that SIRT1 protein binds with USP22 in hiPSC-CMs (Fig. 5H). Besides, downregulation of circ-SIRT1 could inhibit the binding between SIRT1 and USP22 (Fig. 5I). More importantly, either circ-SIRT1 depletion or USP22 knockdown could promote the ubiquitination of SIRT1 (Fig. 5J), indicating that circ-SIRT1 facilitated the USP22-mediated deubiquitination of SIRT1, promoting the stability of SIRT1 protein.

All in all, circ-SIRT1 stabilizes SIRT1 protein via recruiting USP22 to facilitate the deubiquitination of SIRT1 in hiPSC-CMs.

### Circ-SIRT1 inhibits CH via upregulating SIRT1 to enhance autophagy

**Fig. 6:**
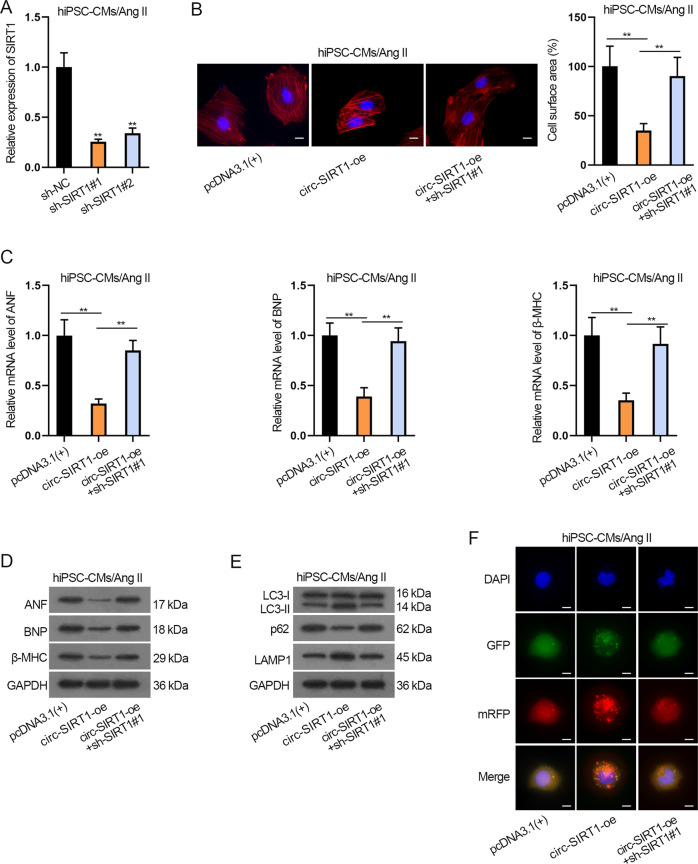
Circ-SIRT1 inhibits CH via upregulating SIRT1 to enhance autophagy. **A** The knockdown efficiency of SIRT1 in Ang II-infused hiPSC-CMs was evaluated via RT-qPCR. *N* = 3. **B** Immunofluorescence staining analysis of the cell surface area after Ang II-infused hiPSC-CMs were transfected with pcDNDA3.1(+), circ-SIRT1-oe, or circ-SIRT1-oe+sh-circ-SIRT1#1. Scale bar, 10 μm. *N* = 3. **C**, **D** RT-qPCR and western blot analyses of the expression of CH markers after Ang II-infused hiPSC-CMs were transfected with pcDNDA3.1(+), circ-SIRT1-oe, or circ-SIRT1-oe+sh-circ-SIRT1#1. *N* = 3. **E**, **F** Autophagy in Ang II-infused hiPSC-CMs of the above-mentioned groups was measured by western blot analysis of autophagy-related proteins and immunofluorescence staining analysis of Ad-GFP-mRFP-LC3. Representative images of fluorescent GFP dots (green), mRFP dots (red), and their merged images are shown. Scale bar, 10 μm. *N* = 3. ^**^P < 0.01 was assessed by one-way ANOVA and Tukey.

At last, examined the influence of circ-SIRT1/SIRT1 axis on CH and autophagy by rescue experiments. First, the knockdown efficiency of SIRT1 was evaluated via RT-qPCR and confirmed that sh-SIRT1#1 presented a better knockdown efficiency in Ang II-treated hiPSC-MCs (Fig. 6A). Thereby, we adopted sh-SIRT1#1 for follow-up experiments. As shown in Fig. 6B and Supplementary Fig. 4D, SIRT1 knockdown reversed the suppressive impact of circ-SIRT1 overexpression on cell surface area and the ratio of well-organized cardiomyocytes of Ang II-treated hiPSC-CMs. Likewise, the suppressive influence of circ-SIRT1 upregulation on the expression of ANF, BNP, and β-MHC was reversed by SIRT1 deficiency in Ang II-infused hiPSC-CMs (Fig. 6C, D and Supplementary Fig. 4E). These data indicated that SIRT1 knockdown countervails the inhibitory effect of circ-SIRT1 on CH. In addition, SIRT1 depletion reverses the promoting effect of circ-SIRT1 overexpression on autophagy in Ang II-treated hiPSC-CMs (Fig. 6E, F and Supplementary Fig. 4F, G). In a word, circ-SIRT1 inhibits CH and promotes autophagy via modulating SIRT1 in Ang II-treated CH cell model.

Besides, we established another CH model in vitro by ISO-treatment to verify the function of circ-SIRT1/SIRT1 on CH via autophagy pathway. The results demonstrated that circ-SIRT1 overexpression reversed the improvement of CH and suppression of autophagy in ISO-treated hiPSC-CMs, and such effect was abrogated by sh-SIRT1#1 or 3-MA (Supplementary Fig. 5A–E).

In addition to in vitro model, we explored the effect of circ-Sirt1 on CH in Ang II-induced in vivo mice model. According to RT-qPCR and western blot analyses, Ang II infusion overtly induced the mRNA and protein levels of ANF, BNP, and β-MHC, and such effect was abolished by circ-Sirt1 overexpression (Supplementary Fig. 6A). Moreover, circ-Sirt1 and Sirt1 were reduced by Ang II infusion in mouse hearts and overexpression of circ-Sirt1 reversed such effect (Supplementary Fig. 6B). Thus, circ-Sirt1 inhibits CH by upregulating Sirt1 in vivo.

## Discussion

It is known that persistent cardiac hypertrophic growth is tightly linked to adverse consequences that may ultimately induce heart failure and also sudden death [38, 39]. Thus, researches are demanded for deeper understanding of the mechanism of its pathology. So far, numerous researches have uncovered a wide range of genes regulating CH, such as HSF1 and EndoA1 [40, 41]. Interestingly, existing evidence has clarified the negative relation of autophagy to CH, that is, promoting autophagy may attenuate CH [16, 31]. SIRT1 is one of the histone deacetylases (HDACs) that bind to protein lysine residues to trigger de-acetylation [42]. Recently, SIRT1 has been manifested to elicit promoting effect on autophagy by de-acetylation of certain genes, such as Beclin1 and ATG9A [14, 15]. Importantly, as previously reported, SIRT1 contributed to the inhibition of CH and SIRT1 was regulated through multiple mechanisms in CH, such as being inhibited by miR-122 or promoted by SP1 and FGF21 [18–20]. CircRNAs are recently discovered to play a part in CH [43]. Although regulation of SIRT1 by circRNAs has been elucidated in some cancers and diseases [25, 27, 44], our study was the first to uncover the circRNA-mediated regulation of SIRT1 in CH. In this study, through bioinformatics and biological assays, we searched out several circRNAs associated with SIRT1. We then proved that circ-SIRT1 and its homologous mouse circ-Sirt1 were low-expressed in Ang II- or ISO-induced CH cell model in vitro and in Ang II infusion-induced CH mice model in vivo, supporting the link between circ-SIRT1 (and circ-Sirt1) and CH. Also, functional assay suggested that circ-SIRT1 and circ-Sirt1 could inhibit CH and improve autophagy in human and mouse cardiomyocytes. Furthermore, by using 3-MA, the autophagy inhibitor, designed rescue assays and proved that inhibiting autophagy can block the inhibitory effect of circ-SIRT1 on CH, suggesting that circ-SIRT1 regulated CH via autophagy pathway. Although circ-SIRT1 was demonstrated to regulated SIRT1 in vascular smooth muscle cells [45], our study first identified that circ-SIRT1 was involved as a negative regulator for CH.

Previous investigations have indicated that circRNAs as ceRNAs were able to regulate multiple cellular behaviors via competing with mRNAs for miRNAs [46, 47]. In this research, we showed that circ-SIRT1 was abundant in the cytoplasm and regulated the luciferase activity of SIRT1 3’UTR but not SIRT1 promoter, indicating circ-SIRT1 might post-transcriptionally regulate SIRT1 expression by acting as a ceRNA of miRNAs in hiPSC-CMs. Expectedly, we predicted and confirmed that miR-3681-3p and miR-5195-3p bound with circ-SIRT1 (or SIRT1) in hiPSC-CMs, and circ-SIRT1 could regulate SIRT1 expression via competitively binding with miR-3681-3p/miR-5195-3p. Previously, miR-3681-3p was documented to participate in acute and chronic hepatitis B virus infection, and miR-5195-3p were supported to suppress the activity of several cancer cells [48, 49], however, our data first established the link between miR-3681-3p/miR-5195-3p with CH. Interestingly, rescue assays confirmed that upregulation of miR-3681-3p and miR-5195-3p could completely reverse the effects of circ-SIRT1 upregulation on SIRT1 mRNA expression but partially on SIRT1 protein expression, suggesting the existence of another pathway in the regulation of SIRT1 protein expression.

By RNA-pulldown and mass spectrometry, we identified that USP22 bound with circ-SIRT1 in cardiomyocytes. USP22 is a deubiquitinating enzyme (DUB) as one member of the ubiquitin-specific protease (USP) superfamily [50]. It has been manifested that SIRT1 protein stability is associated with enzyme-mediated ubiquitination [34, 35]. More importantly, as formally reported, USP22 promotes Sirt1 deubiquitination and stabilization to restrains cell apoptosis [34]. Herein, our study first demonstrated that USP22 also regulated the deubiquitination of SIRT1 in CH and we first discovered that decreased expression of circ-SIRT1 restrained the binding of USP22 with SIRT1, suggesting that circ-SIRT1 promotes the deubiquitination and stabilization of SIRT1 by recruiting USP22 in hiPSC-CMs. Finally, rescue assays delineated that knockdown of SIRT1 could rescue the effect of circ-SIRT1 upregulation on autophagy and CH, confirming the suppressive function of circ-SIRT1/SIRT1 axis in CH.

In conclusion, our study first demonstrated that circ-SIRT1 inhibits CH via upregulating and stabilizing SIRT1 to promote autophagy. This finding provides evidence of the vital function of circ-SIRT1 on regulating autophagy and CH, and sheds novel light on the improvement of CH treatment.

## Supplementary information

Supplementary figure legend

Supplementary Figure 1

Supplementary Figure 2

Supplementary Figure 3

Supplementary Figure 4

Supplementary Figure 5

Supplementary Figure 6

Supplementary Table 1

Supplementary Table 2

Supplementary Table 3

Supplementary File 1

Supplementary File 2

Supplementary File 3

Supplementary File 4

Supplementary File 5

## Data Availability

The data have been provided.
