# Supplementary material for: Circ-SIRT1 inhibits cardiac hypertrophy via activating SIRT1 to promote autophagy
Source: Cell Death Dis. 2021 Nov 10;12(11):1069. doi: 10.1038/s41419-021-04059-y (PMC8580993; doi:10.1038/s41419-021-04059-y)
Supplement: Supplementary file 1 — Supplementary figure legend [file 41419_2021_4059_MOESM1_ESM.docx]

**Supplementary Figure 1**

(A) The effects of Ang II on the ratio of well-organized cardiomyocytes (hiPSC-CMs and H9c2 cells) were evaluated. N=3. (B) Quantification of western blot results of CH markers in TAC group and sham group. N=6 for each group. (C) Ang II serum level in mice of TAC group and sham group was detected. N=6 for each group. (D) RT-qPCR analysis of the efficiency of circ-Sirt1 knockdown in H9c2 cells. N=3. (E) The distribution of circ-SIRT1 or circ-Sirt1 was explored by FISH assay in hiPSC-CMs and H9c2 without or with circ-SIRT1 knockdown and circ-Sirt1 knockdown respectively. Scale bar, 10 μm. N=3. (F) Analysis of the effects of circ-SIRT1 deficiency on the ratio of well-organized cardiomyocytes of hiPSC-CMs. N=3. (G) Western blot quantification of CH-related proteins in hiPSC-CMs transfected sh-NC and sh-circ-SIRT1#1. N=3. (H) Western blot quantification of autophagy-related proteins in hiPSC-CMs transfected with sh-NC or sh-circ-SIRT#1. N=3. (I) Quantification for Figure 2G: Mean numbers of GFP and mRFP dots per cell (left). Mean numbers of autophagosomes represented by yellow dots in merged images and autolysosomes represented by red dots in the merged images per cell (right). N=3. (J) The ratio of well-organized cardiomyocytes of hiPSC-CMs treated without or with Ang II and transfected with pcDNA3.1(+) or circ-SIRT1-oe. N=3. (K) Western blot quantification of CH markers in hiPSC-CMs of different groups. N=3. (L) Western blot quantification of autophagy-related proteins in Ang II-treated hiPSC-CMs transfected with pcDNA3.1(+) or circ-SIRT1-over. N=3. (M) Quantification of Figure 2M: Mean numbers of GFP and mRFP dots per cell (left). Mean numbers of autophagosomes represented by yellow dots in merged images and autolysosomes represented by red dots in the merged images per cell (right). N=3. ^**^P < 0.01 was assessed by Student’s t test for comparison between groups, one-way ANOVA and Dunnett for multiple groups.

**Supplementary Figure 2**

(A) Analysis of the effects of circ-Sirt1 deficiency on cell surface area via immunofluorescence staining (left and middle panels); the effects of circ-Sirt1 knockout on the ratio of well-organized cardiomyocytes (right panel). Scale bar, 10 μm. N=3. (B) The expression of CH markers in H9c2 transfected with sh-NC or sh-circ-Sirt1#1 was examined via RT-qPCR. N=3. (C) The expression of CH markers in H9c2 transfected with sh-NC or sh-circ-Sirt1#1 was examined via western blot analyses (left). Quantification of western blot results (right). N=3. (D) The expression of autophagy-related proteins in H9c2 transfected with sh-NC or sh-circ-Sirt1#1 was examined via western blot analyses (left). Quantification of western blot results (right). N=3. (E) Representative images of fluorescent GFP (green dots), mRFP (red dots), and their merged images are shown (left and middle panels). Scale bar, 10 μm. Mean numbers of autophagosomes represented by yellow dots in merged images and autolysosomes represented by red dots in the merged images per cell (right) were calculated (right panel). N=3. (F) Circ-Sirt1 expression in H9c2 cells treated without or with Ang II and transfected with pcDNA31 (+) or circ-SIRT1-oe was examined via RT-qPCR. N=3. (G) Analysis of the effects of circ-Sirt1 upregulation on cell surface area via immunofluorescence staining, scale bar, 10 μm (left and middle panels); right panel showed the ratio of well-organized cardiomyocytes of H9c2 cells treated without or with Ang II and transfected with pcDNA31(+) or circ-SIRT1-oe. N=3. (H) The expression of CH markers was examined via RT-qPCR in H9c2 treated without or with Ang II and transfected with pcDNA31 (+) or circ-SIRT1-oe. N=3. (I) Western blot bands of CH markers in H9c2 cells treated without or with Ang II and transfected with pcDNA31(+) or circ-SIRT1-oe (left). Quantification of western blot results (right). N=3. (J) The expression of autophagy-related proteins in H9c2 cells of abovementioned groups was examined via western blot analyses (left). Quantification of western blot results (right). N=3. (K) Representative images of fluorescent GFP (green dots), mRFP (red dots), and their merged images are shown (left). Mean numbers of GFP and mRFP dots per H9c2 cell (middle). Scale bar, 10 μm. Mean numbers of autophagosomes represented by yellow dots in merged images and autolysosomes represented by red dots in the merged images per cell (right). N=3. ^**^P < 0.01 was assessed by one-way ANOVA and Tukey.

**Supplementary Figure 3**

(A) Analysis of the ratio of well-organized cardiomyocytes in Ang II-infused hiPSC-CMs which underwent different transfections, including circ-SIRT1 overexpression and circ-SIRT1 overexpression plus 3-MA treatment, taking pcDNA3.1(+) as a negative control. N=3. (B) Western blot results of CH markers in the Ang II-infused hiPSC-CMs transfected with circ-SIRT1-over and circ-SIRT1-over plus 3-MA were quantified. Meantime, pcDNA3.1(+) was used as a negative control. N=3. (C) Quantification of western blot results of autophagy-related proteins (LC3-II/LC3-I, p62, and LAMP1) in the Ang II-infused hiPSC-CMs subjected to different treatments (pcDNA3.1(+), circ-SIRT1-over, and circ-SIRT1-over+3-MA) was shown. N=3. (D) Quantification of Figure 3E: Autophagy-related LC3-GFP-mRFP in the Ang II-infused hiPSC-CMs was subjected to different treatments (pcDNA3.1, circ-SIRT1-over, and circ-SIRT1-over+3-MA). Mean numbers of GFP and mRFP dots per cell (left). Mean numbers of autophagosomes represented by yellow dots in merged images and autolysosomes represented by red dots in the merged images per cell (right). N=3. (E) Circ-SIRT1 overexpression had no marked effect on the luciferase activity of SIRT1 promoter in hiPSC-CMs. N=3. (F) The overexpression efficiency of HIC2 in hiPSC-CMs was verified by qRT-PCR. HIC2 overexpression promoted the luciferase activity of SIRT1 promoter in hiPSC-CMs. HIC2 was used as a positive control for SIRT1 promoter luciferase reporter assay. N=3. (G) The luciferase activity of SIRT1 3’UTR was evaluated in hiPSC-CMs transfected with pcDNA3.1(+) or circ-SIRT1-oe. N=3. (H) RT-qPCR results of the enrichment of SIRT1 mRNA in the pulldown of circ-SIRT1 biotin probe normalized to NC no-biotin probe. N=3. (I) Circ-SIRT1 enrichment in Anti-Ago2 RIP groups compared to that in Anti-IgG RIP controls was analyzed by RT-qPCR. N=3. (J) Quantification of western blot results of SIRT1 protein levels in the hiPSC-CMs transfected with NC mimics or miR-3681-3p mimics (left)/miR-5195-3p mimics (right). N=3. (K) Quantification of western blot results of SIRT1 protein levels in the hiPSC-CMs transfected with sh-NC or sh-circ-SIRT1#1. N=3. ^**^P < 0.01 was assessed by Student’s t test between two groups while one-way ANOVA with Dunnett among multiple groups.

**Supplementary Figure 4**

(A) Quantification of western blot results of SIRT1 protein levels in the hiPSC-CMs transfected with pcDNA3.1(+), circ-SIRT1-over or circ-SIRT1-over+miR-3681-3p mimics+miR-5195-3p mimics. N=3. (B) Quantification of western blot results of USP22 protein levels in the hiPSC-CMs transfected with sh-NC or sh-circ-SIRT1#1. N=3. (C) Quantification of western blot results of USP22 protein levels in the hiPSC-CMs transfected with sh-NC or sh-USP22#1/2 to verify its knockdown efficiency. N=3. (D) Analysis of the ratio of well-organized cardiomyocytes hiPSC-CMs which underwent different treatments, including circ-SIRT1 overexpression or circ-SIRT1 overexpression and SIRT1 knockdown, taking pcDNA3.1(+) as a negative control. N=3. (E-F) Quantification of western blot results of CH markers (ANF, BNP, and β-MHC) and autophagy-related proteins (LC3-II/LC3-I, p62, and LAMP1) in the Ang II-infused hiPSC-CMs which underwent circ-SIRT1 overexpression or circ-SIRT1 overexpression and SIRT1 knockdown were shown respectively. N=3. (G) Quantification of Figure 6F: Mean numbers of GFP and mRFP dots per cell (left). Mean numbers of autophagosomes represented by yellow dots in the merged images and autolysosomes represented by red dots in the merged images per cell (right). N=3. ^**^P < 0.01 was assessed by one-way ANOVA with Dunnett.

**Supplementary Figure 5**

(A) Immunofluorescence analysis of the isoproterenol-treated (ISO-treated) CH model which was treated without (control) or with ISO and co-transfected with pcDNA3.1 (ISO+pcDNA3.1), circ-SIRT1-oer (ISO+circ-SIRT1-oe), circ-SIRT1-oe plus sh-SIRT1#1 (ISO+circ-SIRT1-over+sh-SIRT1#1), or circ-SIRT1-oe plus 3-MA treatment (ISO+circ-SIRT1-over+3-MA). Scale bar, 10 μm (left and middle panels). Right panel shows the ratio of well-organized cardiomyocytes. N=3. (B) The mRNA levels of CH markers were analyzed by qRT-PCR in hiPSC-CMs in the abovementioned groups. N=3. (C) The protein levels of CH markers in hiPSC-CMs of indicated groups were measured via western blot analysis (left). Quantification of western blot results (right). N=3. (D) Autophagy-related proteins were measured via western blot analysis (left). Quantification of western blot results (right). N=3. (E) Representative images of fluorescent GFP (green dots), mRFP (red dots), and their merged images for hiPSC-CMs of indicated groups were shown (left and upper right). Mean numbers of GFP and mRFP dots per cell; Scale bar, 10 μm. Mean numbers of autophagosomes represented by yellow dots in merged images and autolysosomes represented by red dots in the merged images per cell (lower right). N=3. ^**^P < 0.01 was assessed by one-way ANOVA with Tukey.

**Supplementary Figure 6**

(A) The expression and quantification of CH markers in Ang II-treated in vivo model was examined via RT-qPCR and western blot analyses. N=6 for each group. (B) The expression of circ-Sirt1 and Sirt1 and quantification of Sirt1 protein in Ang II-treated in vivo model were examined via RT-qPCR and western blot analyses. N=3. ^**^P < 0.01 was assessed by one-way ANOVA and Tukey.

**Supplementary Table 1**

Recipe of buffers used in the assays.

**Supplementary Table 2**

List of circRNAs whose gene symbol is SIRT1.

**Supplementary Table 3**

Mass spectrometry analysis of the binding proteins of circ-SIRT1 in RNA pulldown assay.
