# Supplementary figures and images for: Circ-SIRT1 inhibits cardiac hypertrophy via activating SIRT1 to promote autophagy
Source: Cell Death Dis. 2021 Nov 10;12(11):1069. doi: 10.1038/s41419-021-04059-y (PMC8580993; doi:10.1038/s41419-021-04059-y)

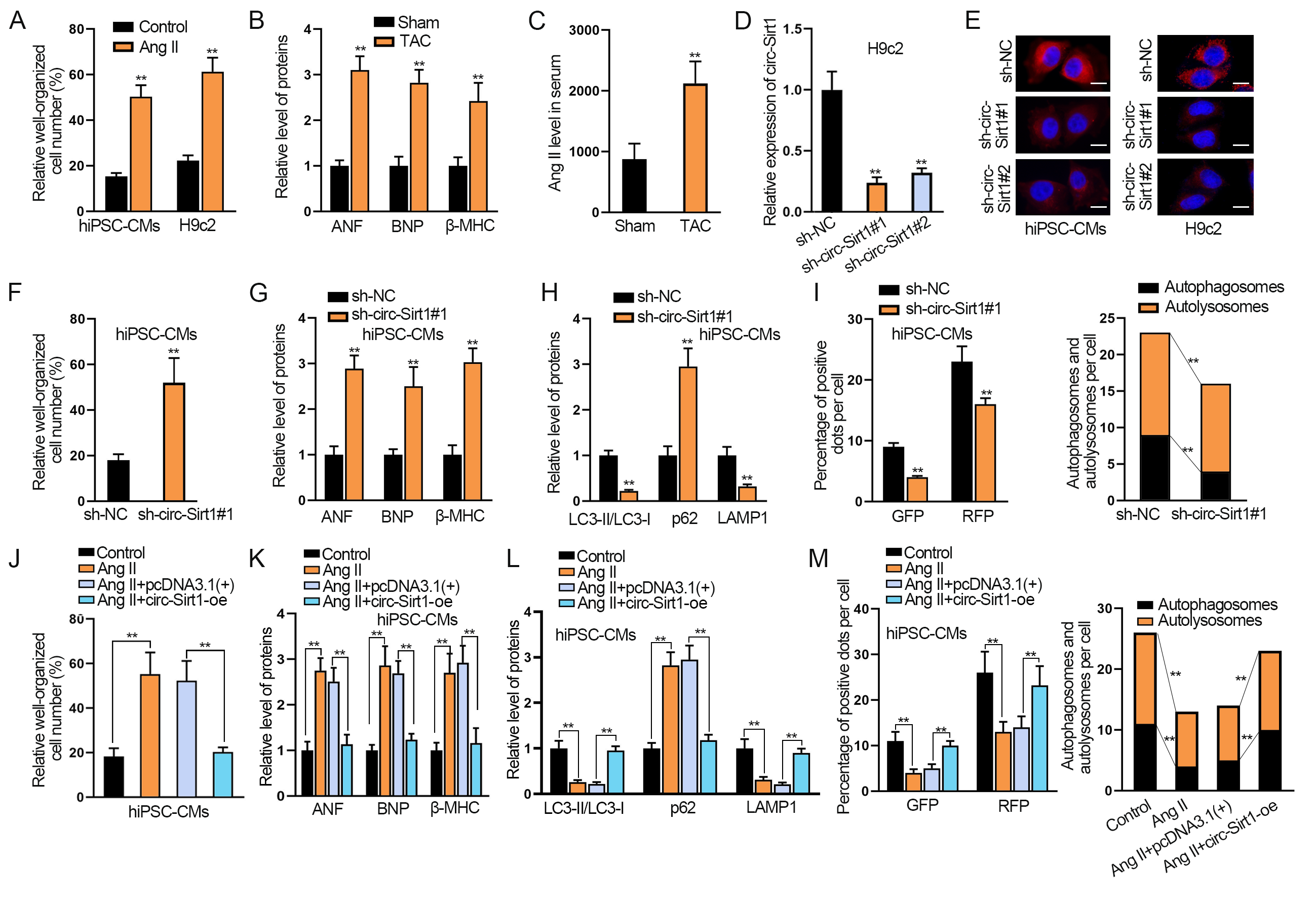

Supplement: Supplementary file 2 — Supplementary Figure 1 [file 41419_2021_4059_MOESM2_ESM.tif]

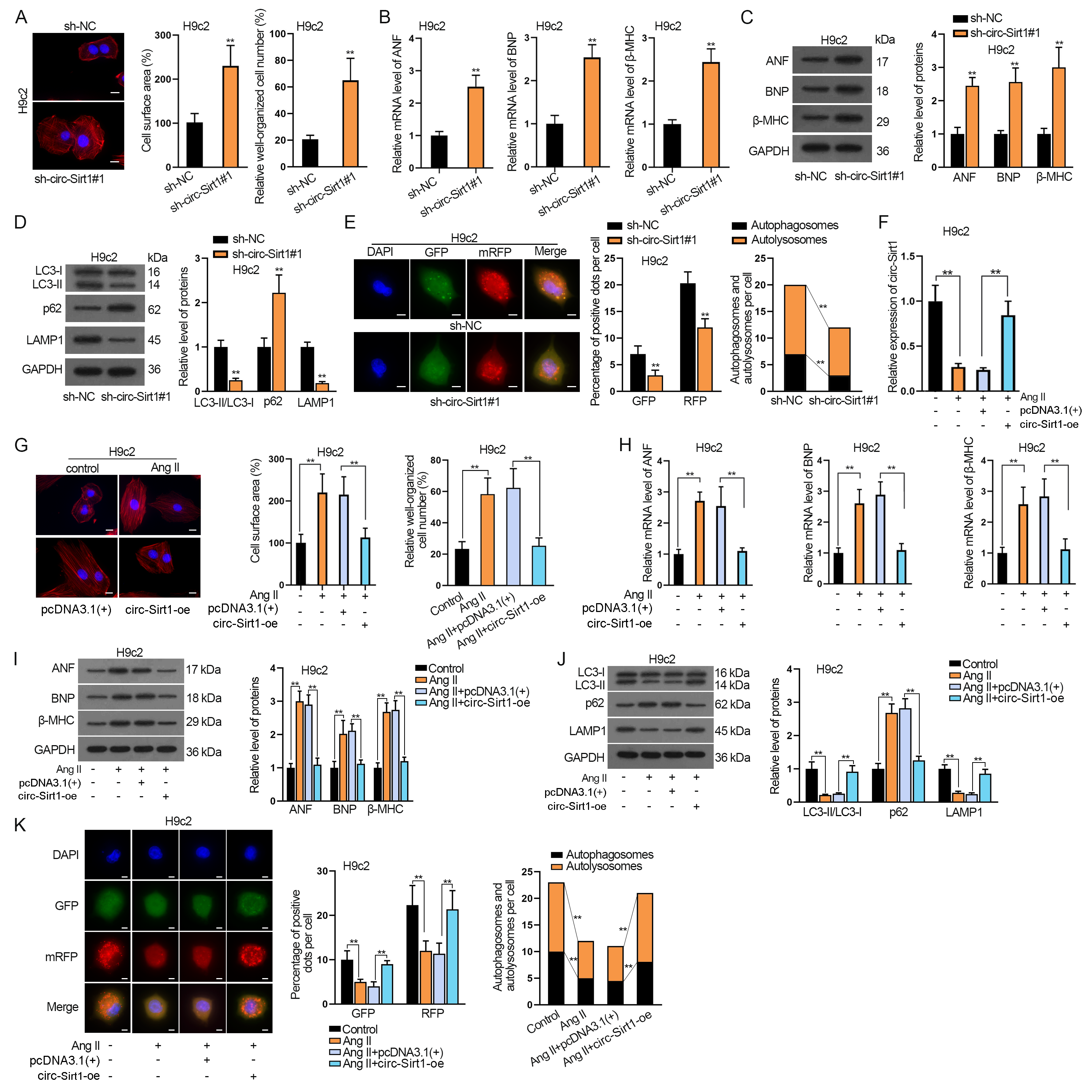

Supplement: Supplementary file 3 — Supplementary Figure 2 [file 41419_2021_4059_MOESM3_ESM.tif]

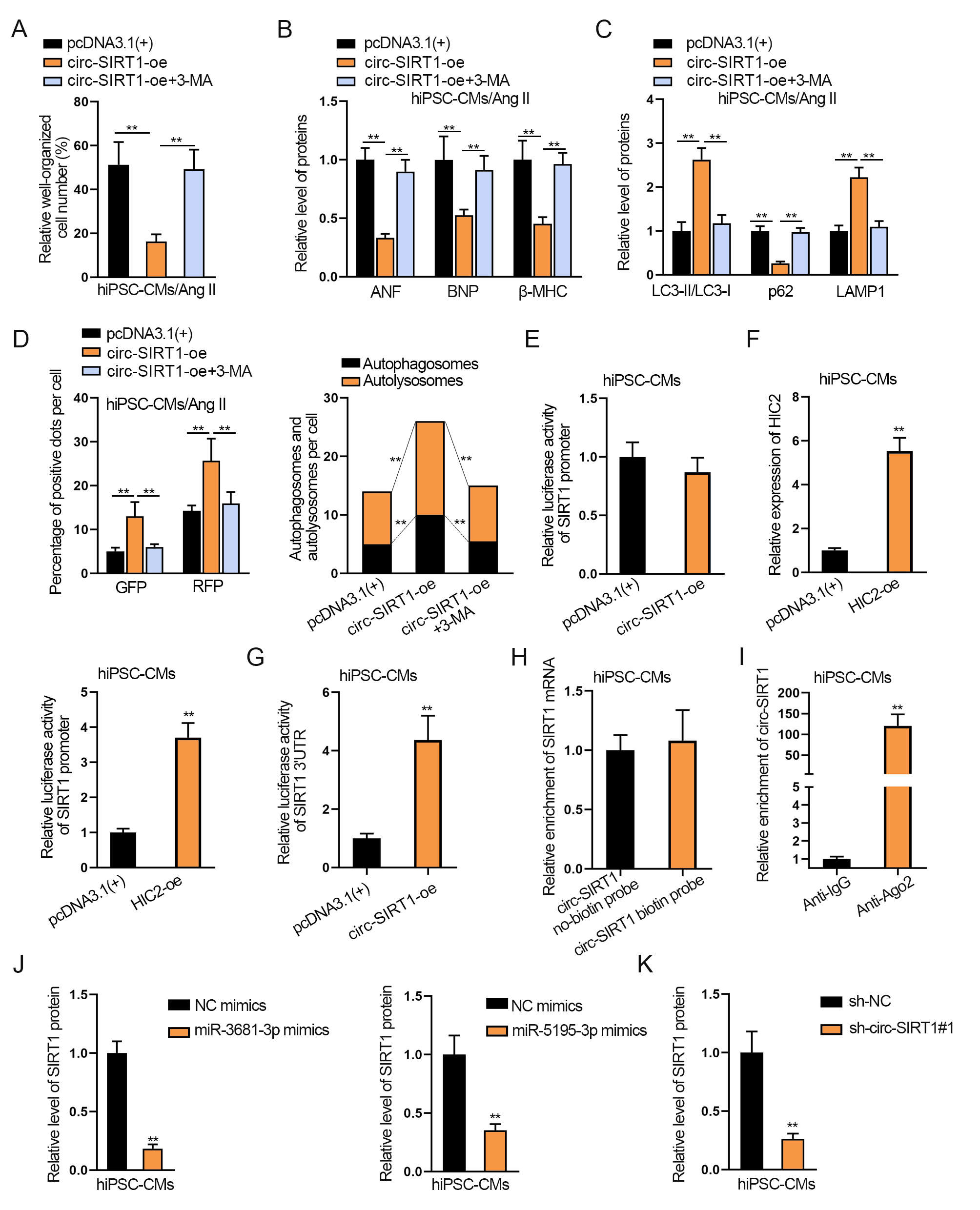

Supplement: Supplementary file 4 — Supplementary Figure 3 [file 41419_2021_4059_MOESM4_ESM.tif]

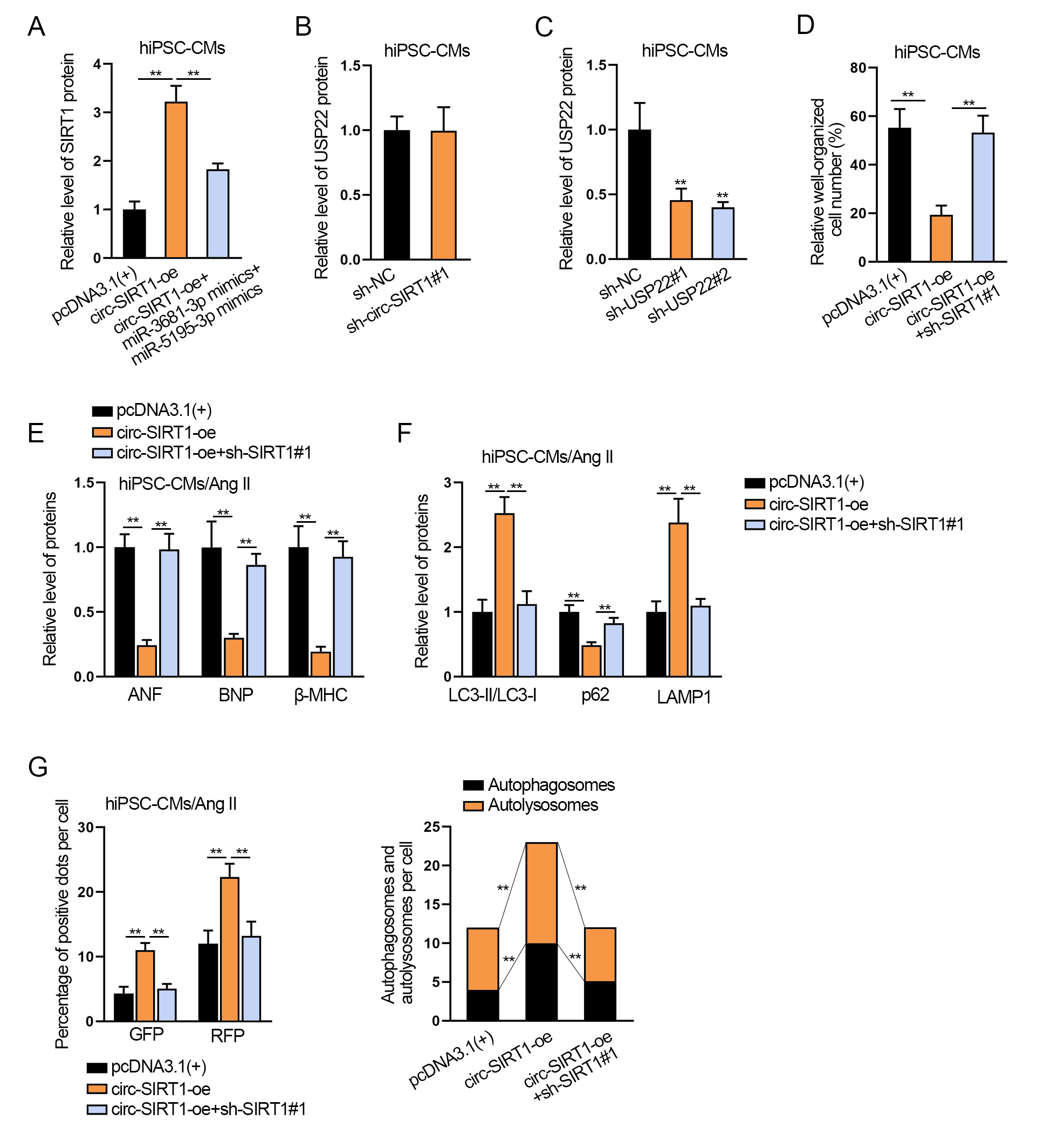

Supplement: Supplementary file 5 — Supplementary Figure 4 [file 41419_2021_4059_MOESM5_ESM.tif]

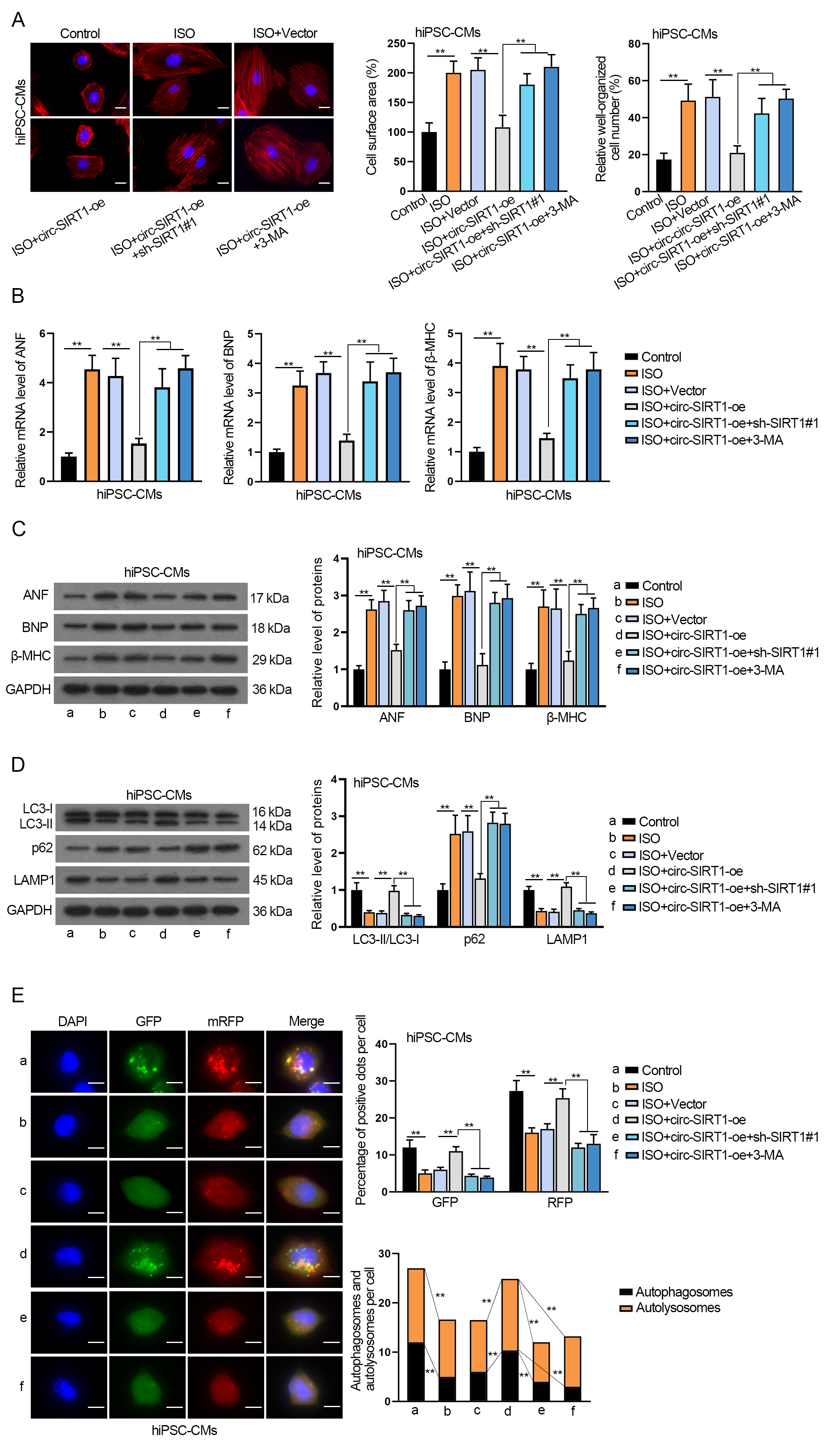

Supplement: Supplementary file 6 — Supplementary Figure 5 [file 41419_2021_4059_MOESM6_ESM.tif]

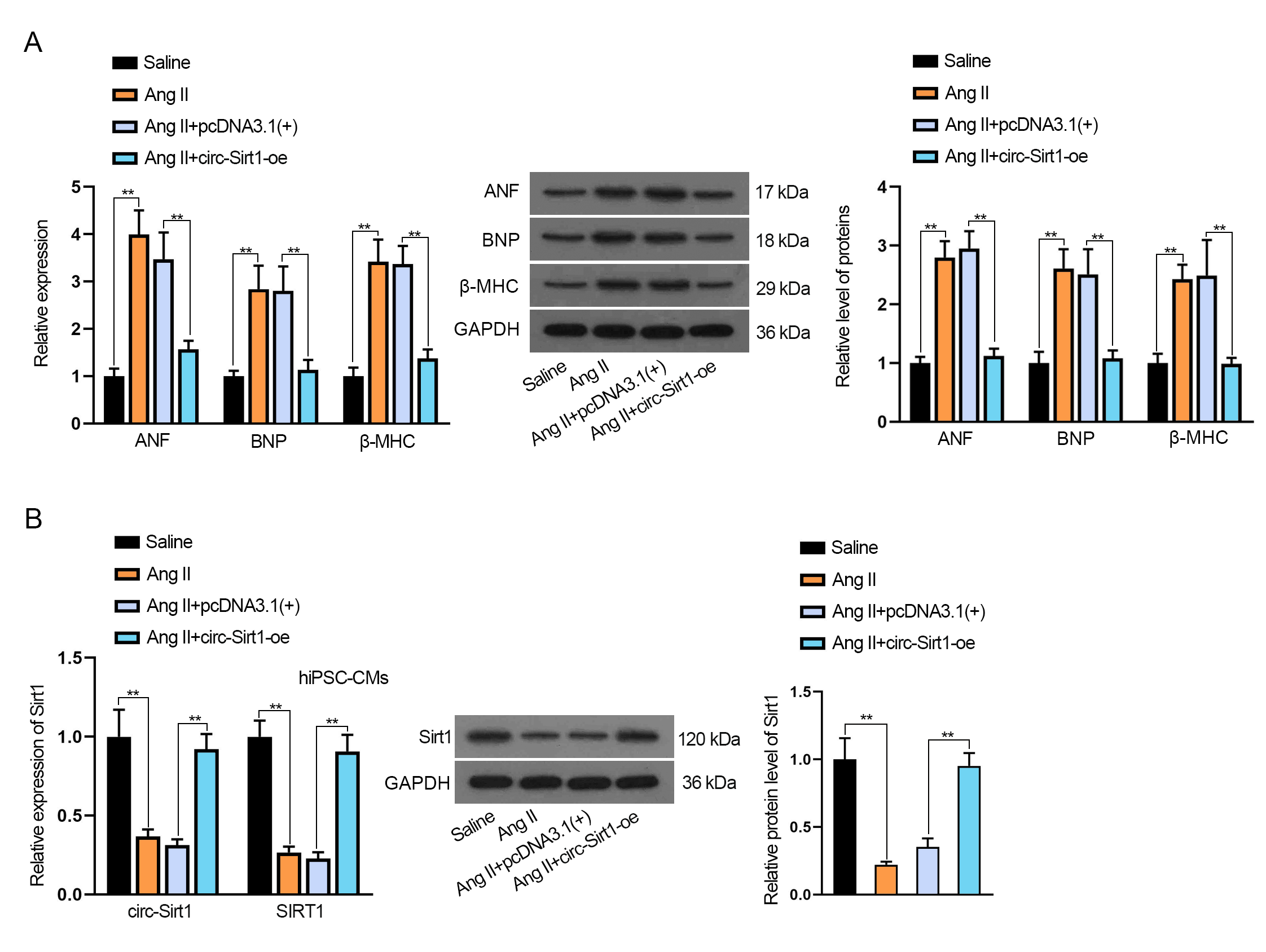

Supplement: Supplementary file 7 — Supplementary Figure 6 [file 41419_2021_4059_MOESM7_ESM.tif]

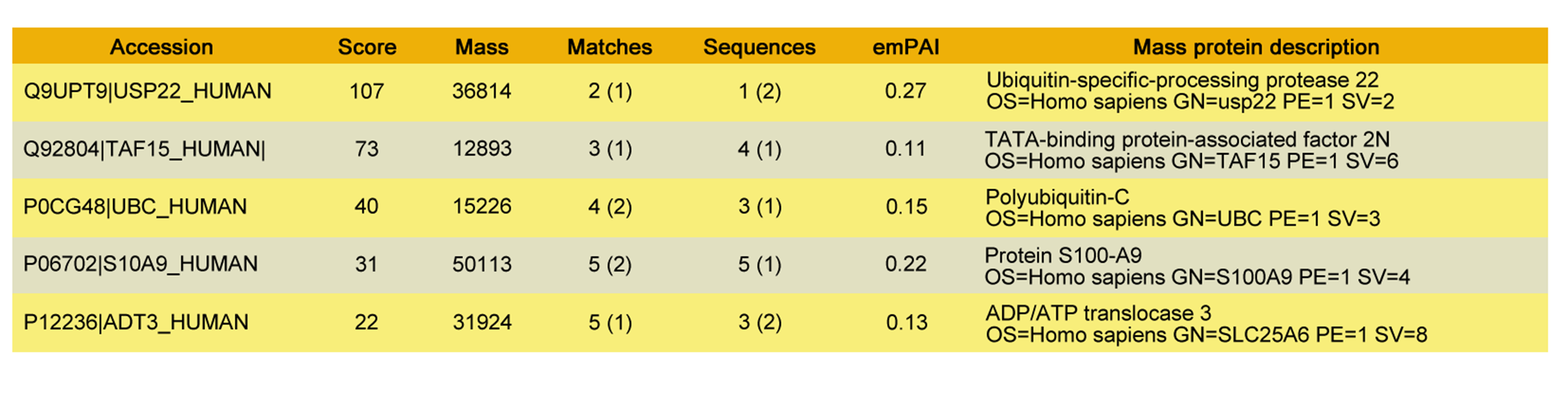

Supplement: Supplementary file 10 — Supplementary Table 3 [file 41419_2021_4059_MOESM10_ESM.tif]

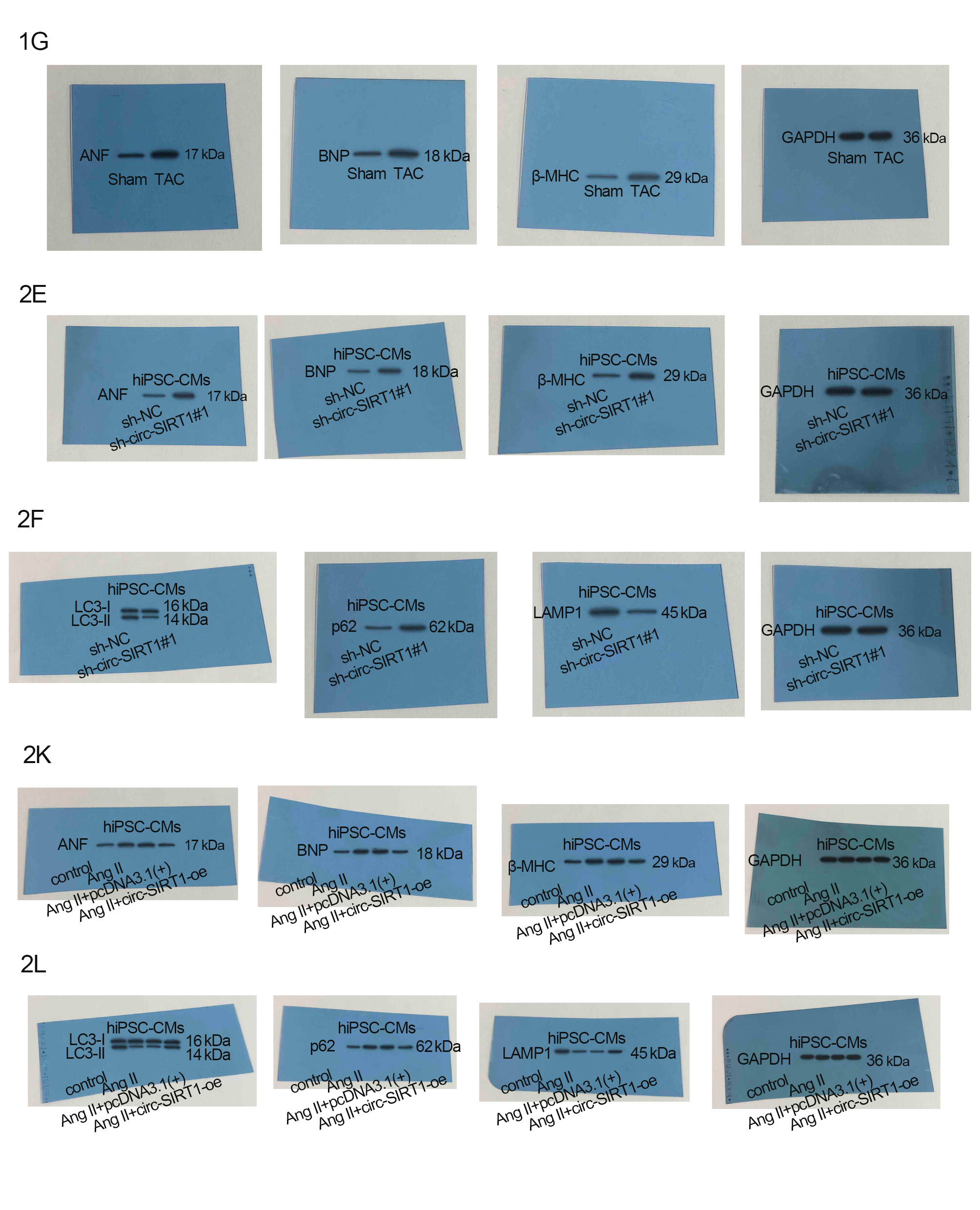

Supplement: Supplementary file 11 — Supplementary File 1 [file 41419_2021_4059_MOESM11_ESM.tif]

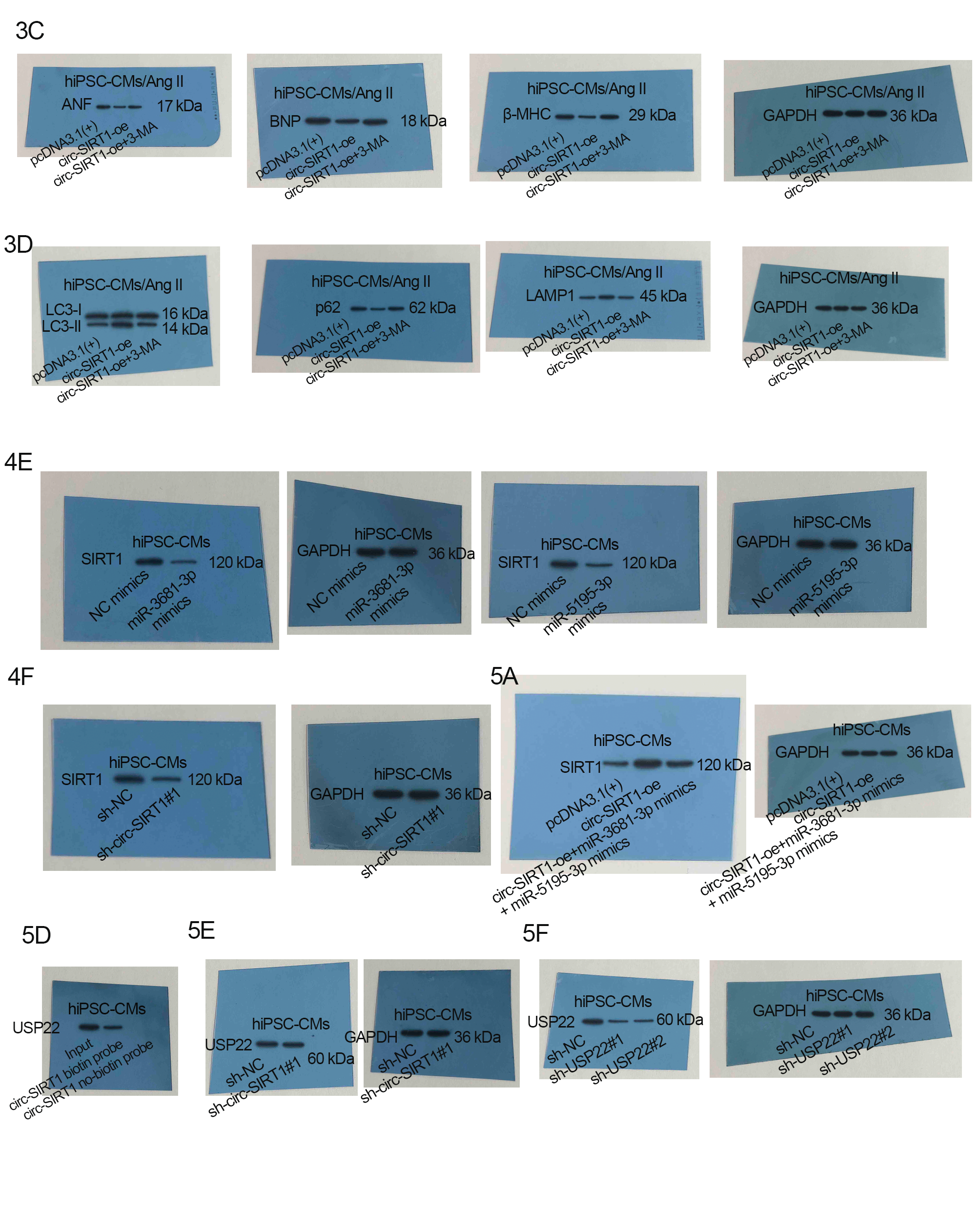

Supplement: Supplementary file 12 — Supplementary File 2 [file 41419_2021_4059_MOESM12_ESM.tif]

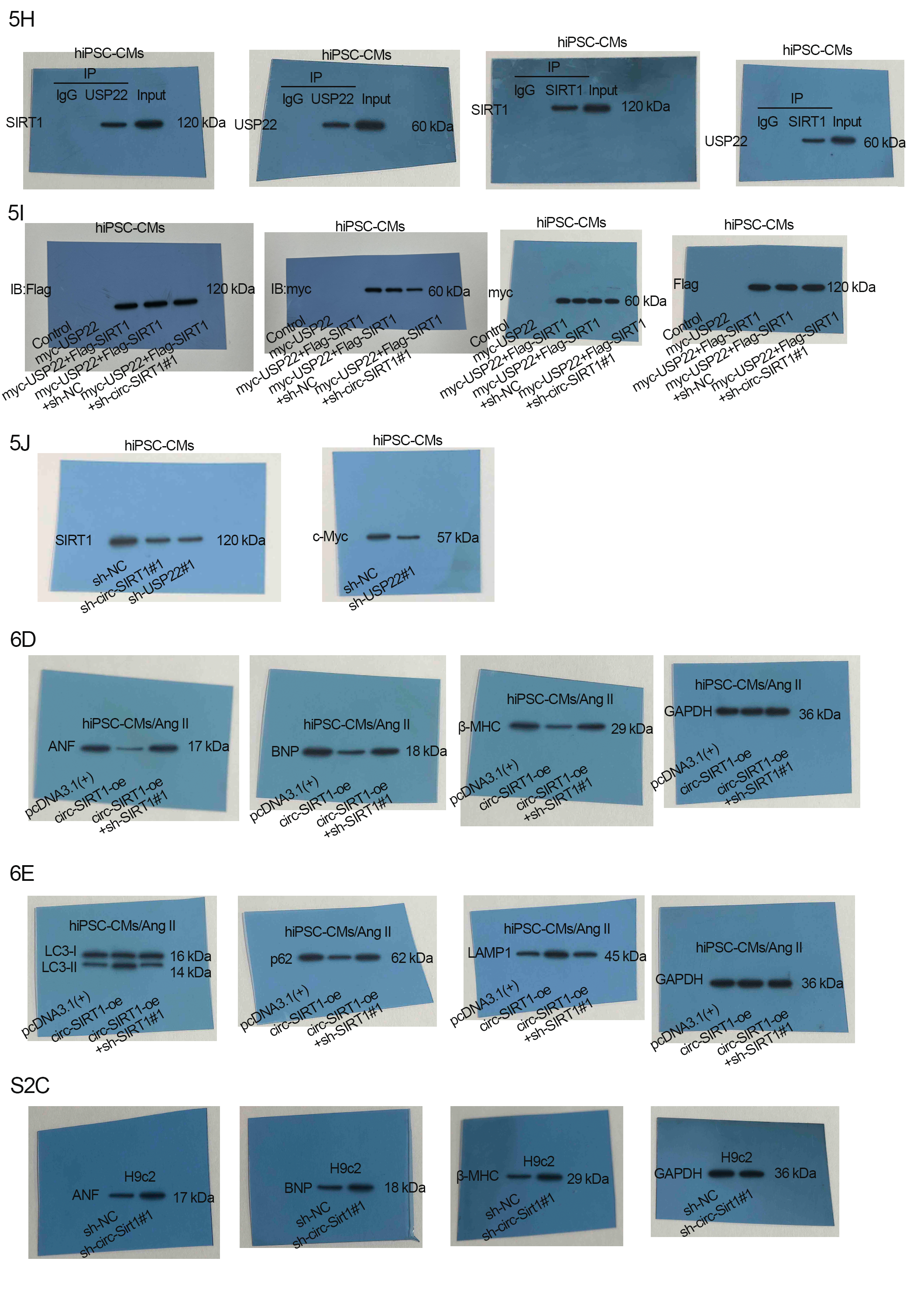

Supplement: Supplementary file 13 — Supplementary File 3 [file 41419_2021_4059_MOESM13_ESM.tif]

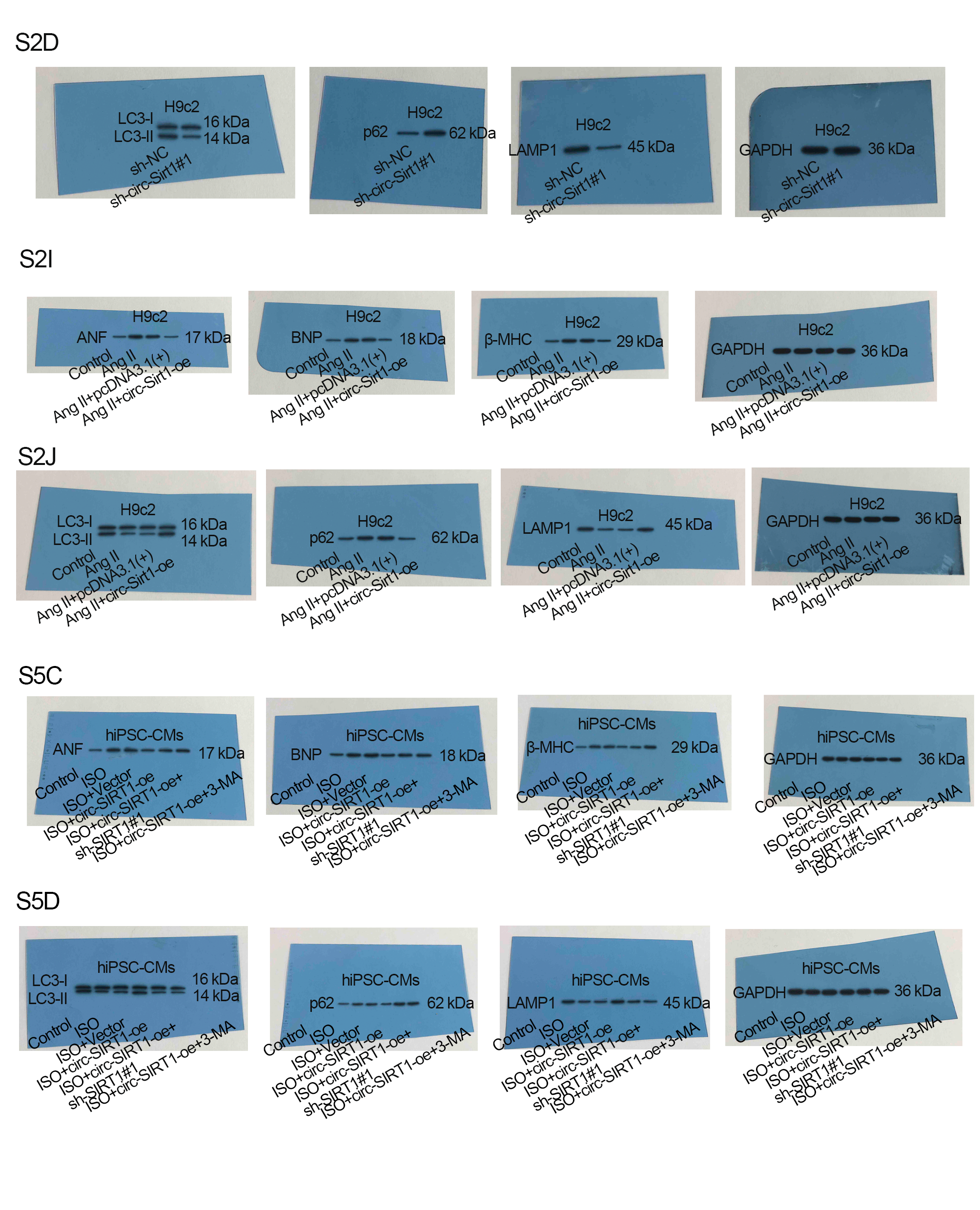

Supplement: Supplementary file 14 — Supplementary File 4 [file 41419_2021_4059_MOESM14_ESM.tif]

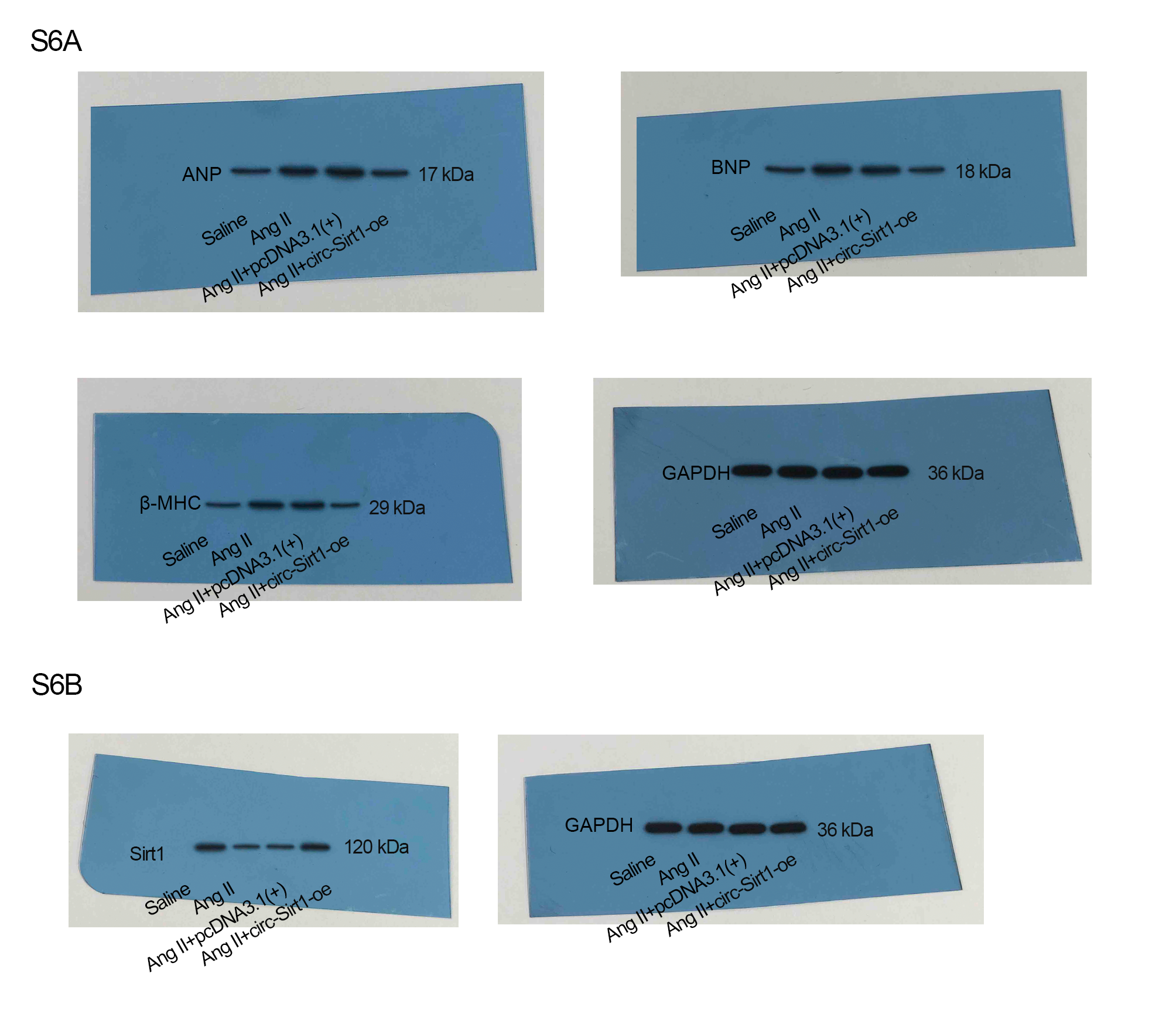

Supplement: Supplementary file 15 — Supplementary File 5 [file 41419_2021_4059_MOESM15_ESM.tif]
