# Supplementary material for: Circ-SIRT1 inhibits cardiac hypertrophy via activating SIRT1 to promote autophagy
Source: Cell Death Dis. 2021 Nov 10;12(11):1069. doi: 10.1038/s41419-021-04059-y (PMC8580993; doi:10.1038/s41419-021-04059-y)
Supplement: Supplementary file 8 — Supplementary Table 1 [file 41419_2021_4059_MOESM8_ESM.docx]

**Supplementary Table 1. Recipe of buffers used in the assays.**

(1)Western blot:

RIPA lysis buffer

| **Components** | **Final Concentration** |
| --- | --- |
| Tris-HCl (pH: 7.4) | 50mM |
| NaCl | 150mM |
| EDTA | 1mM |
| NP40 | 1% |
| Sodium deoxycholate | 0.25% |

Add ddH_2_O and adjust to 100 mL.

(2)CoIP:

IP lysis buffer

| **Components** | **Final Concentration** |
| --- | --- |
| Tris-HCl (pH: 7.4) | 50mM |
| NaCl | 150mM |
| EDTA | 1mM |
| NP40 | 1% |
| Sodium deoxycholate | 0.25% |

Add ddH_2_O and adjust to 100 mL.

4× SDS-loading buffer

| **Components** | **Final Concentration** |
| --- | --- |
| Tris-HCl (pH: 6.8) | 200mM |
| DTT | 200mM |
| SDS | 8% |
| BPB | 0.2% |
| glycerine | 20% |

(3)RNA pulldown assay:

Lysis buffer

| **Components** | **Final Concentration** |
| --- | --- |
| Tris-HCl (pH: 7.4) | 50mM |
| NaCl | 150mM |
| EDTA | 1mM |
| NP40 | 1% |
| Sodium deoxycholate | 0.25% |

Add ddH_2_O and adjust to 100 mL.

RIP washing buffer

| **Components** | **Final Concentration** |
| --- | --- |
| Tris-HCl (pH: 7.4) | 50mM |
| NaCl | 150mM |
| MgCl_2_ | 10mM |
| IGEPAL CA-630 | 0.05% |

Add DEPC-H_2_O and adjust to 100 mL.

(4)FISH assay:

Hybridization buffer

| **Components** | **Final Concentration** |
| --- | --- |
| Formamide deionized | 50% |
| Salt solution | 5× |
| Denhardt’s solution | 5× |
| Dextran sulfate sodium salt | 10% |
| Heparin sodium | 20U/mL |
| SDS | 0.1% |

Add DEPC-H_2_O and adjust to 20 mL.

(5)RIP:

RIP washing buffer

| **Components** | **Final Concentration** |
| --- | --- |
| Tris-HCl (pH: 7.4) | 50mM |
| NaCl | 150mM |
| MgCl_2_ | 10mM |
| IGEPAL CA-630 | 0.05% |

Add DEPC-H_2_O and adjust to 100 mL.
